# Report of the IVth Workshop of the Spanish National Network on Mycotoxins and Toxigenic Fungi and Their Decontamination Processes (MICOFOOD), Held in Pamplona, Spain, 29–31 May 2019

**DOI:** 10.3390/toxins11070415

**Published:** 2019-07-16

**Authors:** Elena González-Peñas, Ariane Vettorazzi, Elena Lizarraga, Amaya Azqueta, Adela López de Cerain

**Affiliations:** 1 Department of Pharmaceutical Technology and Chemistry, School of Pharmacy and Nutrition, Universidad de Navarra, 31008 Pamplona, Spain; 2 Department of Pharmacology and Toxicology, School of Pharmacy and Nutrition, Universidad de Navarra, 31008 Pamplona, Spain; 3 IdiSNA, Navarra Institute for Health Research, 31008 Pamplona, Spain

**Keywords:** MICOFOOD, meeting report, mycotoxins, Spain

## Abstract

The present publication collects the communications presented in the IV Workshop of the Spanish National Network on Mycotoxins and Toxigenic Fungi and their Decontamination Processes (MICOFOOD), held in the School of Pharmacy and Nutrition of the Universidad de Navarra (Pamplona, Spain) from the 29 to the 31 May 2019. More than 70 professionals from academia, the industry and public services have participated. The scientific program included: five sessions: sponsors (presentation and services), toxigenic fungi, toxicology, analysis and control, and reduction and prevention strategies. In total, 18 oral communications and 24 posters were presented. It is worth mentioning the high participation and quality of the communications from PhD students. The invited conference, entitled: “Mycotoxins within the framework of exposure assessment: past present and future”, was given by Dr. Barbara de Santis (Istituto Superiore di Sanità, Rome, Italy). The meeting ended with the roundtable: “From feed to fork: safe food without mycotoxins”, where representatives of feed and agrofood companies and public administrations discussed about the current situation and problems related with mycotoxins. Different prizes were awarded for the best oral presentation (Effect of Staphylococcus xylosus on the growth of toxigenic moulds in meat substrates, by E. Cebrian et al., University of Extremadura), and the best posters (Combined toxicity of aflatoxins and ochratoxin A: A systematic review by M. Alonso-Jaúregui et al., Universidad de Navarra; and Application of natamycin in products affected by toxigenic fungi by Torrijos et al., Universitat de València). The participants had the opportunity to learn about the history and gastronomy of Pamplona. Situated in the north of Spain, Pamplona is a city of Roman origin featuring a large gothic cathedral complex and a Vauban citadel of the 16th century.

## 1. Introduction

Mycotoxins are secondary metabolites produced by fungi and they are considered by the scientific community as one of the most important public health problems (https://www.who.int/news-room/fact-sheets/detail/mycotoxins). The complexity of their influence on human and animal health needs a multidisciplinary approach involving different expertise such as microbiology, toxicology, analytical chemistry, etc. Moreover, collaboration among industries, regulatory agencies and academic scientists is needed in order to achieve the implementation of quality management actions, improve health risk assessment and ensure food quality and safety. In this way, human health can be promoted and protected against mycotoxins.

To achieve these goals, the first Spanish Network of Excellence in the field of mycotoxins was created in 2014 within the projects AGL2014-52648-REDT and AGL2017-90787-REDT of the Spanish Ministry of Economy, Industry and Competitiveness.

The Spanish National Network on Mycotoxins and Toxigenic Fungi and their Decontamination Processes (MICOFOOD) (http://micofood.es) is led by the Laboratory of Food Chemistry and Toxicology of the University of Valencia and comprises the research groups shown in Table 1.

MICOFOOD aims to strengthen the networking among Spanish researchers in the field of toxigenic fungi and mycotoxins. Moreover, its objective is to foster the connection with other similar international networks, industry and public administrations. To this aim, one of the main activities carried out by the Network is the organization of an annual workshop to share knowledge on:

- Characterization of mycotoxin-producing fungi, methodologies for detection and control, biosynthetic pathways and genetic/environmental regulatory mechanisms.
- Development of multi-mycotoxin detection methods in food and biological matrices to be applied for exposure assessment purposes.
- Evaluation of the effects of thermal treatments on the stability and on mycotoxin content during food production, processing and storage.
- Toxicological characterization of individual and combination of mycotoxins.

**Figure 1:**
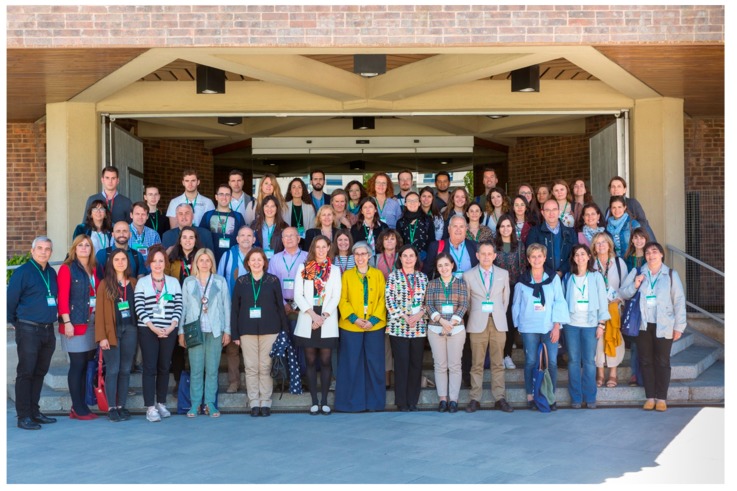
MICOFOOD group.

Four workshops have been organized so far in venues of the different participating research centers: University of Valencia, University of Extremadura, University of Zaragoza and, the most recent one, in the Universidad de Navarra (Figure 1).

## 2. Scientific Committee

Jordi Mañes, University of Valencia, Valencia, Spain

Mónica Fernández-Franzón, University of Valencia, Valencia, Spain

Giuseppe Meca, University of Valencia, Valencia, Spain

Misericordia Jiménez, University of Valencia, Valencia, Spain

Mar Rodríguez, University of Extremadura, Cáceres, Spain

Vicente Sanchís, University of Lleida, Lleida, Spain

Agustín Ariño, University of Zaragoza, Zaragoza, Spain

Covadonga Vázquez, University Complutense of Madrid, Madrid, Spain

Elena González-Peñas, Universidad de Navarra, Pamplona, Spain

Adela López de Cerain, Universidad de Navarra, Pamplona, Spain

Luis González-Candelas, Instituto de Agroquímica y Tecnología de Alimentos (IATA-CSIC), Valencia, Spain

Alberto Cepeda, University of Santiago de Compostela, Lugo, Spain

Francisco Javier Cabañes, Universitat Autònoma de Barcelona, Barcelona, Spain

Ana María García-Campaña, University of Granada, Granada, Spain

## 3. Organizing Committee

Jordi Mañes, University of Valencia, Valencia, Spain

Elena González-Peñas, Universidad de Navarra, Pamplona, Spain

Adela López de Cerain, Universidad de Navarra, Pamplona, Spain

Mónica Fernández-Franzón, University of Valencia, Valencia, Spain

Cristina Juan-García, University of Valencia, Valencia, Spain

Giuseppe Meca, University of Valencia, Valencia, Spain

Lara Manyes, University of Valencia, Valencia, Spain

Yelko Rodríguez-Carrasco, University of Valencia, Valencia, Spain

Dionisia Carballo, University of Valencia, Valencia, Spain

Noelia Pallares, University of Valencia, Valencia, Spain

Juan Manuel Quiles, University of Valencia, Valencia, Spain

Raquel Torrijos, University of Valencia, Valencia, Spain

Tiago De Melo Nazareth, University of Valencia, Valencia, Spain

Manuel Alonso, University of Valencia, Valencia, Spain

Veronica Zingales, University of Valencia, Valencia, Spain

Alessandra Cimbalo, University of Valencia, Valencia, Spain

Ariane Vettorazzi, Universidad de Navarra, Pamplona, Spain

Elena Lizarraga, Universidad de Navarra, Pamplona, Spain

Amaya Azqueta, Universidad de Navarra, Pamplona, Spain

Beatriz Arce-López, Universidad de Navarra, Pamplona, Spain

Borja Muñoz, Universidad de Navarra, Pamplona, Spain

María Alonso, Universidad de Navarra, Pamplona, Spain

Adriana Rodríguez, Universidad de Navarra, Pamplona, Spain

Julen Sanz-Serrano, Universidad de Navarra, Pamplona, Spain

## 4. Oral Presentations

### 4.1. Session I: Sponsors

Chair: J. Mañes (University of Valencia) and E. González-Peñas (Universidad de Navarra)

#### 4.1.1. Mycotoxin Analysis in Food Matrices Using the Miniaturized Ultivo Triple Quadrupole LC/MS System

K. Saitua ^1,^*, J. Morales ^1^ and T. Sosienski ^2^

^1^  Agilent Technologies España, 28232 Las Rozas de Madrid, Spain
^2^  Agilent Technologies. Santa Clara, CA 95051, USA

* Correspondence: koldo_saitua@non.agilent.com

**Abstract:** This presentation demonstrates a sensitive and precise method to analyze 12 mycotoxins in corn and peanut matrices and five mycotoxins in a black pepper matrix in the Ultivo Triple Quadrupole mass spectrometer. Ultivo was designed to save laboratory space, while maintaining the performance required for high-throughput analysis. All mycotoxins could be quantified below the maximum levels defined by the Regulations of the European Commission (EC) No. 1881/2006 and No. 105/2010 in each matrix. Excellent accuracy of the method was achieved in the Ultivo system, with relative standard deviations (RSD%) of <10% at the lowest level of quantification. The combination of the cleanliness of the interferences of the matrix, the chromatography and the recently developed triple quadrupole allows the sensitive and precise detection of mycotoxins.

**Keywords:** LC/MS-MS; miniaturized; triple-quadrupole; mycotoxins; MRL

#### 4.1.2. Mycotoxin Analysis in Food

**F. Rodríguez and N. Filloy**

Phenomenex España, S.L.U.28108 Alcobendas, Madrid, Spain

fernandor@phenomenex.com; noemif@phenomenex.com

**Abstract:** This seminar will describe the main techniques used in the preparation of the sample for the analysis of mycotoxins, describing some of the methodologies for the treatment of the sample and some challenges in its determination. We will visit the determination of aflatoxins, ochratoxin A, fumonisins, trichothecenes and zearalenone in cereals, the preparation of samples and their innovations in analytical methods by LC-MS for the quality control of food samples. In addition, new techniques in the preparation of samples and the technology to face the current challenges in the analysis of multiresidues will be studied.

**Keywords:** introduction and regulatory references; analytical workflow; analytical methods for mycotoxins; multiple waste screening; future perspectives

**Acknowledgments:** We would like to thank especially to Scott Krepich’s Industry Marketing Manager from Phenomenex for his contributions.

#### 4.1.3. A Brief Overview on Waters’ Solutions for Targeted and Untargeted Analysis of Mycotoxins

**P. de la Iglesia**

Waters Cromatografía, 08290 Cerdanyola del Vallès, Barcelona, Spain; pablo_delaiglesia@waters.com

**Abstract:** Mycotoxins are toxic compounds produced by molds or other fungi that can grow on foodstuffs intended for domestic animal or human consumption. Ingestion of food containing only part-per billion concentrations of some mycotoxins may cause health issues. Therefore, sensitive and reliable analytical methods are required to determine mycotoxins in foods and feeds. In this presentation, different chromatographic alternatives aimed at the analysis of mycotoxin will be reviewed. Less selective detectors such as optical fluorescence or single quadrupole MS require the complementary selectivity/specificity provided by immunoaffinity clean-up, especially for the analysis of complex matrices. Additionally, tandem quad MS methods offer more efficient alternatives in terms of analysis time, cost per sample, multi-toxin scope, and sensitivity. Untargeted analysis can be performed with Independent Data Acquisition (IDA) methods supported by high-resolution MS and Ion Mobility Spectrometry, which have proved to be useful to reveal masked mycotoxins in samples, some of them relevant from a toxicological perspective.

**Keywords:** mycotoxins; liquid chromatography; mass spectrometry; ion mobility spectrometry

## 5. Plenary Session Lecture

Chair: E. González-Peñas (Universidad de Navarra)

*Mycotoxins within the framework of exposure assessment: past present and future*

B. De Santis ^1,^*, F. Debegnach ^1^, P. De Santis ^2^ and C. Brera ^1^

^1^  Department of Food Safety, Nutrition and Veterinary Public Health, Chemical Food Safety Unit, Italian National Institute of Health, 00161 Roma, Italy
^2^  Productive and Resilient Farms, Forests and Landscapes, Bioversity International, 00054 Maccarese, Italy

* Correspondence: barbara.desantis@iss.it

**Abstract:** Exposure to mycotoxins has been described since ancient times, with the epidemic intoxications responsible for shingles known as St. Anthony’s fire. These were recorded in the middle age and were attributed to contamination by neurotoxins, ergot alkaloids, produced by Claviceps ssp. Nowadays, with the world running at double speed, we are facing acute episodes of mycotoxicosis in some developing countries, while at the same time, progress is made in the development of a harmonized risk analysis system, where evaluators and risk managers cooperate to develop an effective food safety system. Within the framework of the evaluation of mycotoxins exposure, we value the evolution of analytical techniques, legislation, the actions of the competent authorities, the awareness of various actors of the agrifood sector, and the role over time, of each of the above-mentioned components to the exposure to these natural agricultural contaminants. In recent times, the progress of analytical techniques and other supporting technologies and tools, have led to the capacity either to significantly lower detection limits, to have available the standards of new molecules and metabolites (including isotopically marked standards) and to the possibility of detecting multiple toxins within a single sample. Exposure assessment requires that the limits of detection be as low as possible, while at the same time maintaining satisfactory levels of precision. Quality of data plays a central role in the EFSA forum, where particular attention is given to continuity in data collection of contaminants and to the development of harmonized methodologies. Mycotoxin exposure assessment methodologies are mainly focused on the deterministic approach but probabilistic calculations are becoming a supportive tool for refinement of exposure scenarios. Mycotoxins represent an extremely important issue within the program DG SANTE (Directorate General of Health and Food Safety of the European Commission). In the Commission agenda on agricultural contaminants, both classical and emerging mycotoxins are considered, and during the last 15 years, in addition to the definition of maximum limits the commission has also dealt with applying legislative measures and defining the standards to be applied by the actors of the agro-food chain to allow a correct and effective application of prevention measures. Despite the efforts made, there are still sensitive issues, which are not dealt with by the authorities: the role of the scientific community is to be vigilant and to work to fill the existing gaps.

**Keywords:** mycotoxins; exposure assessment; data quality; risk analysis

## 6. Oral Communications

### 6.1. Session II: Toxigenic Fungi

Chair: C. Vázquez (University Complutense of Madrid) and M. Jiménez (University of Valencia)

#### 6.1.1. Genome Resequencing of Ochratoxigenic and Non Ochratoxigenic Strains of *Aspergillus Carbonarius*

G. Castellá ^1,^*, M.R. Bragulat ^1^, L. Puig ^1^, W. Sanseverino ^2^ and F.J. Cabañes ^1^

^1^  Veterinary Mycology Group, Department of Animal Health and Anatomy, Veterinary Faculty, Universitat Autònoma de Barcelona, 08193 Bellaterra, Barcelona, Spain
^2^  Sequentia Biotech S.L., 08193 Bellaterra, Barcelona, Spain

* Correspondence: Gemma.Castella@uab.cat

**Abstract:** Ochratoxin A (OTA) is a mycotoxin that can be found in a variety of common foods and beverages. This mycotoxin is produced by several species of *Penicillium* and *Aspergillus* among which *Aspergillus carbonarius* is recognized as the main OTA source on grapes and derived products [1]. OTA production is a very consistent property of *A. carbonarius* and nearly 100% of the isolates of this species produce OTA [2]. In the present study, we present the genome resequencing of an OTA producer strain of *A. carbonarius* and three atypical non-OTA producing strains [3]. These strains were sequenced using Illumina technology and compared with the genome reference Acv3. We performed some new specific bioinformatics analyses in genes involved in OTA biosynthesis. We focused these analyses on nonsense and missense mutation detection, and also in to identify large DNA deletions in the genome of the three non-OTA producing strains. The OTA-producer strain showed variants in *AcOTApks*, *AcOTAnrps*, *AcOTAbZIP* and *AcOTAp450*. In atoxigenic strains, five common missense variants in *AcOTApks* gene were found. Although some gaps of more than 1,000 bp were identified in nonochratoxigenic strains, no large deletions in functional genes related with OTA production were found. Moreover, the expression of five genes of the putative OTA biosynthetic cluster was downregulated under OTA-inducing conditions in the nonochratoxigenic strains.

**References**:

1. Cabañes, F.J.; Bragulat, M.R. *Ochratoxin A in Profiling and Speciation Aspergillus in the Genomic Era*; Varga, J., Samson, R.A., Eds.; Wageningen Academic Publishers: Wageningen, The Netherlands. 2008; pp. 57–70.
2. Cabañes, F.J.; Bragulat, M.R.; Castellá, G. Characterization of nonochratoxigenic strains of *Aspergillus carbonarius* from grapes. *Food Microbiol.* **2013**, *36*, 135–141.
3. Castellá, G.; Bragulat, M.R.; Puig, L.; Sanseverino, W.; Cabañes, F.J. Genomic diversity in ochratoxigenic and non ochratoxigenic strains of *Aspergillus carbonarius*. *Sci. Rep.* **2018**, *8*, 5439.

**Keywords:***Aspergillus carbonarius*; comparative genomics; genome; ochratoxin A; resequencing

**Acknowledgments:** This research was supported by the *Ministerio de Economía y Competitividad* of the Spanish Government (AGL2014-52516-R).

#### 6.1.2. Characterization of Two Genes Putatively Involved in Griseofulvin Biosynthesis in *Penicillium Griseofulvum*

A. Cometto ^1^, D. Spadaro ^1^, L. González-Candelas ^2^ and A.-R. Ballester ^2,^*

^1^  Department of Agricultural, Forestry and Food Sciences, University of Torino, 10195 Grugliasco, Italy
^2^  Food Biotechnology Department, Instituto de Agroquímica y Tecnología de Alimentos (IATA-CSIC), 46980 Paterna, Valencia, Spain

* Correspondence: ballesterar@iata.csic.es

**Abstract:***Penicillium griseofulvum* is worldwide distributed and has been isolated from fruits, decayed plants, cereal grains and animal feed. *P. griseofulvum* has been associated with blue mould decay during storage of apple fruits, which is considered as one of the most important postharvest diseases of pome fruits worldwide. This fungus is also known to produce a wide array of important secondary metabolites, including griseofulvin and patulin. The griseofulvin biosynthetic gene cluster, consisting of 13 putative genes, was originally described in *P. aethiopicum* (Chooi et al., 2010). Banani et al. (2016) reported the complete genome sequence of *P. griseofulvum* strain PG3 and described the gene cluster responsible for the synthesis of griseofulvin, comparing it with the griseofulvin gene cluster in *P. aethiopicum.* The putative transcription factor gsfR1 is located inside the griseofulvin biosynthetic cluster, while, gsfR2 is located outside the gene cluster. The aim of this work was the characterization, through *A. tumefaciens* mediated transformation, of the putative transcription factors GsfR1 and GsfR2, involved in the regulation of griseofulvin production in *P. griseofulvum*. These putative transcription factors have not been characterized yet and they could play an important role in the regulation of this compound. This work will enable to underline the role of the putative transcription factors encoded by gsfR1 and gsfR2 genes in griseofulvin biosynthesis.

**Keywords:***Penicillium griseofulvum*; Griseofulvin; knockout mutants

**Acknowledgments:** this work was supported by the Spanish Ministry of Science, Innovation and Universities (AGL2017-88120-R and RTI2018-093392-A-I00 (MCIU/AEI/FEDER, UE), by the LIFE financial instrument of the European Union (Contract LIFE13 ENV/HR/000580) and by Fondazione Cassa di Risparmio di Cuneo (SMART APPLE project).

**References**:

1. Banani, H.; Marcet-Houben, M.; Ballester, A.-R.; Abbruscato, P.; González-Candelas, L.; Gabaldón, T.; Spadaro, D. Genome sequencing and secondary metabolism of the postharvest pathogen *Penicillium griseofulvum*. *BMC Genom.* **2016**, *17*, 19.
2. Chooi, Y.-H.; Cacho, R.; Tang, Y. Identification of the viridicatumtoxin and griseofulvin gene clusters from *Penicillium aethiopicum*. *Chem. Biol.* **2010**, *17*, 483–494.

### 6.2. Session III: Toxicology

Chair: A. López de Cerain (Universidad de Navarra) and M. Fernández-Franzón (University of Valencia)

#### 6.2.1. Effect of Enniatins in Mitochondrial Processes of Female Rats

A. Cimbalo *, L. Bayarri, M.J. Ruiz, M. Alonso and L. Manyes

Laboratory of Food Chemistry and Toxicology, Faculty of Pharmacy, University of Valencia, 46100 Burjassot, Spain

* Correspondence: Alessandra.cimbalo@uv.es

**Abstract:** Enniatins (ENs) are cyclic depsipeptide mycotoxins produced by a group of *Fusarium* fungi. They have been found as natural contaminants in food and feed and they can severely interfere with human health. In particular, it has been shown they affect mitochondrial processes by modifying oxidative phosphorylation. The aim of the present study was to evaluate toxicological effects of ENs in the electron transport chain in vivo. A total of 14 female, two-month-old Wistar rats were employed divided in three groups: control, medium and high exposure. The four rats of the control group were exposed to the vehicle (PBS) by esophageal intubation, while the five of the treated ones were intoxicated with medium concentrations: single dose of EN A 256, EN A1 353, EN B 540, EN B1 296 g/mL; and other five with the higher ones: single dose of EN A 513, EN A1 706, EN B 1021, EN B1 593 g/mL. They were sacrificed after eight hours of exposure. The rats’ liver, stomach and kidneys were analysed. RNA was extracted from tissues and transcriptional analysis was carried out by qPCR using the SYBR^TM^ Green method. Four genes of the electronic transport chain were selected by using KEGG (Kyoto Encyclopedia of Genes and Genomes) Pathway Database. In particular, ND1, CO1, ATP5, Sdha and S18 as a reference gene. Gene expression was calculated by StepOnePlus software. Statistical analysis was performed by applying the Student’s *t*-test. Results in the liver showed considerable group variability in gene expression among all the animals employed. In the stomach, the main differences in gene expression have been reported between control and medium-dose treated rats, divided into upregulation and downregulation for all the analysed genes. In kidneys, ND1 and CO1 expression was clearly affected by the medium concentration used while Sdha expression varies according to the concentration employed. No changes have been observed for ATP5. To sum up, despite the high concentration of ENs used, no significant changes in gene expression were observed.

**Keywords:** oxidative phosphorylation; gene expression; in vivo; qPCR

**Acknowledgments:** This work was supported by the Spanish Ministry of Science, Innovation and Universities (AGL 2016-77610R) and the Generalitat valenciana (GVPROMETEO2018-126).

#### 6.2.2. High Overexpression of MTRF1a Protein after Bauvericin and Enniantin b Mixture Exposure in Vitro

M. Alonso-Garrido ^1,^*, I.E. Pralea^2^, C. Iuga ^2^, M.J. Ruiz ^1^, G. Font ^1^ and L. Manyes ^1^

^1^  Laboratory of Food Chemistry and Toxicology, Faculty of Pharmacy, University of Valencia, 46100 Burjassot, Valencia, Spain
^2^  Department of Proteomics and Metabolomics, MedFuture Research Center for Advanced Medicine, Iuliu, Hatieganu University of Medicine and Pharmacy, 400012 Cluj-Napoca, Romania

* Correspondence: manuel.alonso-garrido@uv.es

**Abstract:** Beauvericin and enniatin B are two emergent mycotoxins from *Fusarium* fungi that are frequently detected concomitantly in cereals and cereal-based products. While in vitro they have shown ionophoric activity and subsequent mitochondriotoxic properties among others, slight adverse effects in vivo, such as loss of weight, have been found. At transcriptomic level in Jurkat lymphoblastoid T-cell line, both revealed a similar downregulation pattern affecting most of the genes involved in the electron transport chain pathway. In order to explore the adverse outcome pathway that leads to loss of homeostasis, it was proposed to investigate the changes in mitochondrial protein expression in Jurkat cells. The chosen combination of beauvericin and enniatin B was 1:1 at three different doses: 0.01–0.1–0.5 µM. Control cells were exposed to 0.5% dimethylsulfoxide (DMSO). Cells were treated during 24 h and then mitochondria were extracted. Three biological replicates and technical duplicates from each treatment were injected in a Nano-LC-Q-TOF ultra-definition MS^E^ and raw data were processed using Swiss-Prot database. After comparing the control and the three doses results from the 1821 proteins identified and quantified, 340 proteins were selected using the parameters: max fold change ≥ 1.5 and ANOVA *p*-value ≤ 0.05. These selected proteins were analyzed by different bioinformatics tools for proteomics data interpretation. The most overrepresented pathways using Reactome version 66 were the citric acid cycle and respiratory electron transport, mitochondrial protein import and respiratory electron transport, ATP synthesis by chemiosmotic coupling, and heat production by uncoupling proteins. PANTHER (Protein Analysis Through Evolutionary Relationship) version 14.1 indicated protein transmembrane import into intracellular organelle as the most overrepresented biological process and disulfide oxidoreductase activity as molecular function. Moreover, Jurkat cells exposed to beauvericin and enniatin B low concentration mixture highly overexpress mtRF1a, mitochondrial peptide chain release factor 1-like showing versus control a fold change > 34 in all conditions.

**Keywords:** mycotoxin; mitochondria; proteomics; Jurkat

**Acknowledgments:** This work was supported by the Spanish Ministry of Science, Innovation and Universities (AGL 2016-77610R and BES-2017-081328).

#### 6.2.3. Mycotoxins Passage Across Blood–Brain Barrier in Vitro: A Metabolomics Approach

N. Pallarés *, M. Alonso-Garrido, M.J. Frangiamone Ruiz and L. Manyes

Laboratory of Food Chemistry and Toxicology, Faculty of Pharmacy, University of Valencia, 46100 Burjassot, Spain

* Correspondence: noelia.pallares@uv.es

**Abstract:** Metabolomics is an emerging ‘omics’ discipline that measures the dynamic responses of the metabolome to various stimuli. Mycotoxins are ingested through contaminated food and feed and are able to reach the bloodstream and cross the blood–brain barrier (BBB). Among more than 400 mycotoxins identified, beauvericin and enniatins are cytotoxic compounds, with the ability to alter intracellular ion homeostasis; aflatoxins are classified as human carcinogens, their toxic effects include genotoxicity, teratogenicity and immunosuppressive activity; ochratoxin is a potent nephrotoxic and zearalenone shows endocrine disrupting effects. BBB is a permeable but selective cell wall that defends the brain from toxic compounds by separating brain extracellular fluid from blood. The aim of this study is to investigate mycotoxins BBB passage, individually and mixed, and their neurotoxicity mechanisms from a metabolomics perspective. The BBB in vitro model was prepared using ECV 304 endothelial human cells over an insert and C6 glial rat cells on the bottom of the well, both plated at a density of 50,000 cel/mL. Transendothelial electric resistance was measured every day after day 4 to ensure the integrity of BBB. At around day 9 of coculture, coinciding with the highest integrity of the barrier, the experiment was carried out. ECV 304 differentiated endothelial human cells were exposed to beauvericin, zearalenone, enniatins, aflatoxins and ochratoxin, individually or in combination, at concentration of 100 nM for 2 h. Apical and basal media were collected separately for each insert-well for the extraction of extracellular metabolites. The extraction method distinguishes between lipidic and aqueous phases. Extracts were lyophilized (aqueous phase) or dried under N2 stream (lipidic phase) and maintained at −20 °C before resuspension in mobile phase for injection. Finally, the extracellular metabolites were profiled using an Agilent 6540 Ultra High Definition UHD Accurate Mass Quadrupole Q-TOF LC/MS instrument. Results were analyzed by MassHunter software and metabolites were identified via METLIN (Metabolite and Chemical Entity Database).

**Keywords:** Endothelial human cells ECV 304; C6; extracellular metabolites; Quadrupole-Time of Flight (Q-TOF); Liquid Chromatography/Mass Spectrometry (LC/MS)

**Acknowledgments:** this work was supported by the Spanish Ministry of Science, Innovation and Universities (AGL 2016-77610R and BES-2017-081328) and Universitat de València (UV-INV-PREDOC16F1-384781).

#### 6.2.4. Altered Base Detection by the Comet Assay

D. Muruzábal ^1,^*, J. Sanz-Serrano ^1^, S. Sauvaigo ^2^, B. Treillard ^2^, A. López de Cerain ^1,3^, A. Vettorazzi ^1,3^ and A. Azqueta ^1,3^

^1^  Department of Pharmacology and Toxicology, School of Pharmacy and Nutrition, Universidad de Navarra, 31008 Pamplona, Spain
^2^  LXRepair, Parvis Louis Néel, 38040 Grenoble, France
^3^  IdiSNA, Institute for Health Research, 31008 Pamplona, Spain

* Correspondence: dmuruzabal@alumni.unav.es

**Abstract:** The comet assay is included in the EFSA guidance on genotoxicity testing strategies for chemical substances present in food and feed, including mycotoxins. The comet assay on its standard-alkaline version is able to detect strand breaks and alkali-labile sites at the single-cell level. The assay has been combined with DNA repair enzymes to augment its sensitivity towards other lesions. The aim of this work was to optimize the conditions and evaluate the performance of different enzymes in combination with the comet assay to detect altered bases. Different DNA repair enzymes were used in combination with the comet assay in TK-6 cells treated with non-cytotoxic concentrations of methyl methanesulphonate (MMS) and ethyl methanesulphonate (EMS), to induce alkylation damage, or potassium bromate (KBrO_3_) and H_2_O_2_, to induce oxidized bases. The enzyme-modified comet assay was also applied in cells treated with cytotoxic concentrations of Triton X-100. The DNA repair enzyme activity was also tested on GlycoSpot biochips by LXRepair (France). Results showed that the DNA repair enzymes used were able to detect different levels of the oxidized and alkylated bases in a specific manner. None of the enzymes showed activity in untreated or Triton X-100-treated cells. Moreover, the expected specificity of the enzymes was detected in the GlycoSpot biochips. The enzyme-modified comet assay using a combination of DNA repair enzymes is a valuable tool, not only to study the potential genotoxic effect of compounds in food, but also to elucidate the mechanism of action of genotoxic compounds detected in food, such as some mycotoxins.

**Keywords:** enzyme-modified comet assay; DNA repair enzymes; altered bases

**Acknowledgments:** Spanish Ministry of Economy, Industry and Competitiveness (BIOGENSA, AGL2015-70640-R). D.M thanks the Government of Navarra and the Ministry of Economy, Industry and Competitiveness for the pre-doctoral grants received. J.S. thanks the *Asociación de Amigos* of the Universidad de Navarra and the Government of Navarra for the pre-doctoral grants received. A.A. thanks the Ministry of Economy, Industry and Competitiveness (*Ramón y Cajal* programme, RYC-2013-14370) of the Spanish Government for personal support.

### 6.3. Session IV: Analysis and Control

Chair: F.J. Cabañes (Universitat Autònoma de Barcelona) and A.M. García-Campaña (University of Granada)

#### 6.3.1. Validation of an Analytical Method for the Multidetection and Quantification of Mycotoxins in Human Plasma Using LC-MS/MS

B. Arce-López *, E. Lizarraga, M. Flores-Flores, Á. Irigoyen and E. González-Peñas

Navarra, Research group MITOX, Pharmaceutical Technology and Chemistry Department, School of Pharmacy and Nutrition, Universidad de Navarra, 31008 Pamplona, Spain

* Correspondence: barce@alumni.unav.es

**Abstract:** Mycotoxins are secondary fungi metabolites that represent a significant worldwide problem. Their presence in food causes several adverse effects on humans. Exposure to mycotoxins could be by different routes and, often, more than one mycotoxin is found simultaneously in the diet. Few analytical studies in human biological fluids have been carried out, and they are mainly limited to the most studied mycotoxins. Therefore, it is necessary to develop methods that allow the study of human’s exposure. The objective of this work is to develop a high resolution liquid chromatography method coupled to a mass detector (triple quadrupole) (LC-ESI-MS/MS) for the extraction and quantification of 19 mycotoxins: deoxynivalenol, deepoxy-deoxynivalenol, aflatoxins (B1, B2, G1, G2, M1), T-2 and HT-2 toxins, ochratoxins A and B, zearalenone, sterigmatocystin, nivalenol, 3-acetyldeoxynivalenol, 15-acetyldeoxynivalenol, neosolaniol, diacetoxyscirpenol and fusarenon-X. Sample preparation is simple and it is achieved using Agilent Captiva cartridges EMR (Enhanced Matrix Removal) lipids (3 mL) and acetonitrile (1% formic acid). Validation was carried out taking into account the following parameters: limits of detection (LOD) and quantification, linearity, precision (repeatability and intermediate precision), recovery and matrix effect (both in intermediate precision conditions) and stability. Mean values for recovery were between 68.23% for sterigmatocystin and 97.90% for diacetoxyscirpenol (RDS ≤ 15% in all the cases). The matrix effect was low and ranged from 76.5 for the sterigmatocystin to 109.3 for the ochratoxin B with a RSD (%) of less than 15% for all mycotoxins. LOD values range between 0.08 ng/mL for aflatoxin B1 and 2.7 ng/mL for HT-2 toxin, except 9.1 ng/mL for nivalenol. In consequence, this methodology is adequate for its use in human biomonitoring of mycotoxins.

**Keywords:** mycotoxins; mutidetection; human plasma; LC-MS/MS; human biomonitoring 

**Acknowledgments:** this work was supported by the Spanish *Ministerio de Economía, Industria y Competitividad, Agencia Estatal de Investigación* (MultiMYCOtox project AGL2017-85732-R, MINECO/AEI/FEDER, UE).

#### 6.3.2. Essential Oils as a Sustainable Method to Control *Aspergillus Flavus*

M. García-Díaz ^1,^*, A. Medina-Vaya ^2^, B. Patiño ^1^, E. García-Cela ^2^, C. Vázquez ^1^ and J. Gil-Serna ^1^

^1^  Genetics, Physiology and Microbiology Department, University Complutense of Madrid, 28040 Madrid, Spain
^2^  Applied Mycology Group, Cranfield University, MK43 Cranfield, UK

* Correspondence: martga43@ucm.es

**Abstract:** Aflatoxins (AFs) are the most important mycotoxins due to the risk they pose to food safety, and *Aspergillus flavus* is the most relevant producing species. Currently, essential oils (EOs) are considered efficient sustainable methods to control fungal growth since their potent fungicide activity is well known. In the present work, the in vitro effect of seven EOs extracted from aromatic plants from two different years (2015 and 2016) was evaluated regarding *A. flavus* growth and its ability to produce AFs. The experiments were performed in CYA (Czapek Yeast Extract Agar) medium supplemented with EOs at concentrations from 10 to 1000 µg/mL. All EOs analyzed reduced fungal growth and AFL production to some extent, although those from winter savory (*Satureja montana*) and origanum (*Oreganum virens*) were by far the most effective ones. Subsequently, the effect of both EOs was evaluated in a wide range of water activities (a_w_ 0.94-0.96-0.98) and concentrations (350, 700 y 1000 µg/mL) using two *A. flavus* isolates (A7 y A10). Fungal growth was measured by turbidimetry using a Bioscreen C analyzer and AFL concentration was determined by HPLC. Winter savory EO was able to retard fungal growth at all concentrations tested and its effect was more significant than that observed using origanum EO. In both cases, the effect was related to a_w_ and growth was reduced more at a_w_ 0.94. No statistically significant differences were found regarding both isolates. In general, AFL production was reduced at a_w_ 0.94 and 0.98 although the presence of EOs at low levels at a_w_ 0.96 supposed an induction in AFL production.

**Keywords:** aflatoxins; *Aspergillus flavus*; essential oil; *Satureja montana*; *Origanum virens*

**Acknowledgments:** work financed by MINECO (AGL2014-53928-C2-2-R).

#### 6.3.3. Dietary Exposure to Mycotoxins through Alcoholic and Nonalcoholic Beverages Consumption

D. Carballo ^1,^*, G. Font ^2^, E. Ferrer ^2^, H. Berrada ^2^

^1^  Faculty of Agricultural Science, National University of Asunción, 2160 San Lorenzo, Paraguay
^2^  Laboratory of Food Chemistry and Toxicology, Faculty of Pharmacy, University of Valencia, 46100 Burjassot, Valencia, Spain

* Correspondence: dio.carballo@gmail.com

**Abstract:** The introduction of health warnings has boosted the consumption of beverages with low alcohol contents; however beer and wine are heavily consumed beverages in the European Union. Mycotoxins are secondary metabolites of fungi commonly present in fruits (grape and apple) and cereals (barley and corn) raw material used in the production of wine and beer. The objective of this study was to analyze the presence of 30 mycotoxins; AFB_1_, AFB_2_, AFG_1_, AFG_2_, AOH, AME, ENA, ENA_1_, ENB, ENB_1_, BEA, FB_1_, FB_2_, OTA, STG, DON, 3-ADON, 15-ADON, NIV, NEO, DAS, FUS-X, PAT, ZEA, α-ZAL, β-ZAL, αZOL, β-ZOL, T-2 and HT-2 in 110 samples of alcoholic and nonalcoholic beverages such as beer, wine, cider and cava, to evaluate the exposure to mycotoxins through beverages consumption in the Valencian population. The validated method based on dispersive liquid–liquid microextraction and chromatographic methods coupled to tandem mass spectrometry was applied. The results showed that beer samples were the most contaminated samples, even at concentrations ranging from 0.24 to 54.76 µg/L. A significant incidence of alternariol was found in wine reaching concentration levels of up to 43.48 µg/L. Patulin and ochratoxin A were the most frequently detected mycotoxins in cava and cider samples with incidences of 26% and 40%, respectively. Ochratoxin A was found in one wine sample exceeding the maximum level established by the EU. A combined assessment of exposure was performed, based on the sum of mycotoxin concentrations detected in the same sample to approach the dietary exposure magnitude to mycotoxins through these beverages. The risk associated with mycotoxin levels obtained in the analysed beverages was not significant.

**Keywords:** mycotoxins; LC-MS/MS; GC-MS/MS

**Acknowledgments:** Spanish Ministry of Economy and Competitiveness (AGL2016-77610-R) and Government Scholarship program “Carlos Antonio López–Paraguay”.

#### 6.3.4. Effect of Spices on Ochratoxin A Production During the Processing of Dry-Cured Fermented Sausages

M. Álvarez *, I. Martín, P. Padilla, A. Rodríguez, J.J. Córdoba, M. J. Andrade

Food Hygiene and Safety, Meat and Meat Products Research Institute, Faculty of Veterinary Science, University of Extremadura, 10003 Cáceres, Spain

* Correspondence: maalvarezr@unex.es

**Abstract:** Despite the fact that the antibacterial activity of spices commonly added to dry-cured fermented sausages is well known, their antagonistic activity against ochratoxigenic moulds in these products has not been studied yet. The aim of the study was to determine the efficacy of the addition of oregano, rosemary and thyme on controlling the ochratoxin A (OTA) production of *Penicillium nordicum* in a dry-cured fermented sausages-based medium over a period of 14 days at 12 °C. All the spices were separately added at 0.2% (w/w) to the medium. In order to simulate the processing of dry-cured fermented sausages, a piece of casing was placed on the surface of each one of the media. An antifungal preparation was used as a positive control. At the end of the incubation period, mycelium samples were taken for extracting OTA, which was subsequently quantified by uHPLC-MS/MS QqQ. Results showed significant reductions of OTA in the dry-cured fermented sausages-based media containing oregano, rosemary or their combination with the antifungal preparation. Nevertheless, the addition of the antifungal preparation at subinhibitory concentrations for the *P. nordicum* growth did not reduce the OTA production with respect to the control samples. Consequently, the addition of oregano or rosemary during the manufacturing of dry-cured fermented sausages could be used as a strategy to control the hazard related to the presence of OTA in this kind of meat products.

**Keywords:***Penicillium nordicum;* ochratoxin A; dry-cured fermented sausages; spices; antagonistic activity

**Acknowledgments:** this work has been supported by IB16045 and GR18056 projects (Junta de Extremadura-Consejería de Economía e Infraestructuras-, Fondo Europeo de Desarrollo Regional-“Una manera de hacer Europa”). M. Álvarez is recipient of a pre-doctoral fellowship (BES-2017-081340) from the Spanish Ministry of Economy, Industry and Competitiveness.

#### 6.3.5. Evaluation of the Presence of Mycotoxins in Bile of Dairy Cows

C. Nebot ^1,^*, C. M. Franco ^1^, E.M. Peris Montesa ^2^, L. Quintela ^2^ and A.Cepeda ^1^

^1^  Hygiene, Food Inspection and Control Laboratory, Faculty of Veterinary Medicine, University of Santiago de Compostela, 27002 Lugo, Spain
^2^  Department of Animal Pathology, Reproduction and Obstetrics, Faculty of Veterinary Medicine, University of Santiago de Compostela, 27002 Lugo, Spain

* Correspondence: carolina.nebot@usc.es

**Abstract:** In the last 15 years, Galician dairy cattle has lost 30% of its population, even though, in 2017, Galician dairy cattle accounted for 42% of the Spanish dairy cattle. In the last years, a progressive and worrisome decline in fertility in the breeding of dairy cattle, the data indicate a decrease of approximately 20% in the last 30 years. Factors such as genetics, the absence of well-being, inadequate nutrition, poor reproductive management and the increase of reproductive diseases may be the cause of this decline. A diet with toxic substances of environmental origin can have effects on follicular fluid and uterine content and therefore pose harmful consequences on reproductive efficiency. Mycotoxins are a clear example of toxic substances of environmental origin that can be ingested by the animal through the feeding provided. For all this and given the importance of reproductive efficiency in milk production influencing the profitability of farms, the objective of this research is to conduct a preliminary study to estimate the prevalence of mycotoxins in bile. Fifty samples of bile were taken from animals slaughtered in a Lugo slaughterhouse and the presence of 10 mycotoxins was evaluated with a method developed by the laboratory based on immunoaffinity columns and subsequent detection of mycotoxins by HPLC-MS/MS. None of the bile samples analyzed showed residues of aflatoxin B1, B2, G1 and G2, deoxynivalenol, diacetoxyscirpenol, fumonisin B2, ochratoxin A, T2-toxin and zearalenone. These results agree with data provided by the Galician Association of Compound Food Manufacturers since they also did not detect the presence of the mycotoxins investigated in the raw materials evaluated.

**Keywords:** dairy cows; bile; HPLC-MS/MS

**Acknowledgments:** We would like to thank Phenomenex and especially Biopharm for their support in the method development.

### 6.4. Session V. Reduction and Prevention Strategies

Chair: A. Ariño (University of Zaragoza) and M. Rodriguez (University of Extremadura)

#### 6.4.1. Effect of *Staphylococcus Xylosus* on the Growth of Toxigenic Moulds in Meat Substrates

E. Cebrián *, F. Núñez, A. Alía, E. Bermúdez and M. Rodríguez

Food Hygiene and Safety, Meat and Meat Products Research Institute, Faculty of Veterinary Science, University of Extremadura, 10003 Cáceres, Spain

* Correspondence: evcebrianc@unex.es

**Abstract:** During the ripening process of dry-cured meat products, the ecological conditions sometimes favour the growth of moulds on their surface. Some are producers of mycotoxins being an issue for food safety. One strategy to control this hazard is the use of microorganisms that are commonly found in these meat products, such as cocci Gram +, catalase + belong to the genus *Staphylococcus*, one of the major microbial groups in this type of products throughout the processing. The aim of this work was to evaluate the effect of a strain of *Staphylococcus xylosus*, isolated from dry-cured ham, on the growth of moulds producer of ochratoxin A (OTA), aflatoxins (AFs) and cyclopiazonic acid (CPA) at three different temperatures (15, 20 and 25 °C) in a culture medium elaborated with dry-fermented sausage. For this, 10 strains belonging to five species of moulds were used: *Penicillium nordicum*, *P. verrucosum*, *P. griseovulfum*, *Aspergillus flavus* and *A. parasiticus*. The diameter of the colonies was measured daily in two perpendicular directions for 30 days. The results showed that the presence of *S. xylosus* significantly decrease the growth of all moulds at the three study temperatures from the beginning of their growth. The greatest reduction of the growth rate was observed for strains of OTA-producing and AFs-producing moulds at 20 °C. However, for ACP-producing moulds, the effect was bigger at 25 °C. These results are promising, although more extensive studies are needed, especially in the evaluation of the effect on the production of the different mycotoxins before being proposed as a protective culture.

**Keywords:** Toxigenic moulds; *Staphylococcus xylosus* growth; dry-cured meat products

**Acknowledgments:** This work has been financed by the Spanish Ministry of Economy and Competitiveness, Government of Extremadura and FEDER (AGL2016-80209-P, GR18056).

#### 6.4.2. Antifungal Activity of Fermented Mustard Extracts with Lactic Acid Bacteria against *Fusarium* spp.

R. Torrijos ^1,^*, T.M. Nazareth ^1,2^, F.J. Martí ^1^, J.M. Quiles ^1^, C. Luz ^1^, J. Mañes ^1^ and G. Meca ^1^

^1^  Departamento de Medicina Preventiva, Facultad de Farmacia, University of Valencia, 46100 Burjassot, Valencia, Spain
^2^  School of Life Science, Pontifícia Universidade Católica do Paraná, 1155 Curitiba, Brasil

* Correspondence: Raquel.Torrijos@uv.es

**Abstract:** The contamination of food products by toxigenic fungi is currently a challenge in food safety. Lactic acid bacteria (LAB) are commonly used in the food industry for their metabolic properties, being producers of substances of interest such as bacteriocins, organic acids and bioactive peptides. In the present study, two types of aqueous extracts made from yellow mustard flour (YMF) and Oriental mustard flour (OMF) fermented with nine different LAB isolated from tomato and sourdough were evaluated against fungi of the *Fusarium* genus. First, a qualitative evaluation of the antifungal activity of the extracts in solid medium (PDA) was carried out. The BALs that showed antifungal potential were selected to establish the minimum inhibitory concentration (MIC) and the minimum fungicide concentration (MFC). At the same time, the extracts were characterized, evaluating the antioxidant capacity by the 2,2’-azino-bis(3-ethylbenzothiazoline-6-sulphonic acid (ABTS) test and the content of phenolic acids previous extraction by QuEChERS (Quick, Easy, Cheap, Effective, Rugged and Safe) methodology and identification by Liquid Chomatography-Electrospray/Mass Spectrometry Quadropole Time of Flight (LC-ESI/MS Q-TOF). Four strains of *Lactobacillus plantarum* were selected for their antifungal properties. The fermented YMF extracts showed a higher antifungal activity, with MIC values between 7.8 and 31.3 g/L and MFC values between 15.6 and 62.5 g/L. The fermentation increased the antioxidant capacity of the extracts tested, with a higher content of antioxidant compounds in the extracts made from YMF. Regarding the content of phenolic compounds, nine phenolic acids with powerful antimicrobial capacity were identified. Future assays will proceed to study the protein and peptide content, focusing on their respective antimicrobial activity.

**Keywords:** antifungal activity; *Fusarium* spp.; lactic acid bacteria; mustard

**Acknowledgments:** The research was supported by the Spanish Ministry of Economy and Competitiveness (AGL2016-77610R), the project Prometeo (2018/216) and by the pre-PhD program of the Spanish Ministry of Science, Innovation and Universities (FPU17/06104).

#### 6.4.3. Use of Natural Compounds to Reduce the Growth of *Aspergillus Flavus* and the Production of Aflatoxin B1 in Almonds

T.M. Nazareth ^1,2,^*, R. Torrijos ^1^, K.C.P. Bocate ^2^, C. Luz ^1^, J.M. Quiles ^1^, F.B. Luciano ^2^, J. Mañes ^1^, G. Meca ^1^

^1^  Laboratory of Food Chemistry and Toxicology, Faculty of Pharmacy, University of Valencia, 46100 Burjassot, Valencia, Spain
^2^  School of Life Science, Pontifícia Universidade Católica do Paraná, 1155 Curitiba, Brasil

* Correspondence: tiago@uv.es

**Abstract:** The objective of this study was to evaluate the effect of yellow mustard flour (YMF), oriental mustard flour (OMF), allyl isothiocyanate (AITC) and a fermented of lactic acid bacteria (LAB) against the growth of *Aspergillus flavus* and the production of aflatoxin B1 (AFB_1_) in almonds. The almonds were treated by fumigation in direct (YMF and LAB) and indirect contact (OMF and AITC), according to the volatilization capacity of the natural compounds evaluated. On days 7 and 15, microbial counts were performed and the mycotoxin production was quantified by HPLC-MS/MS. The results showed that the use of AITC and OMF inhibited the growth and production of mycotoxins when doses higher than 5 μL/L and 2g/L were applied, respectively. The application of YMF and LAB resulted in a significant increase in the fungal population as well as the concentration of AFB_1_. In conclusion, only the natural treatments containing AITC in its formulation had antifungal and antimycotoxigenic effects. In addition, the results evidenced in this work could help the development of new technologies for the conservation and higher safety of nuts.

**Keywords:** Allylisothiocyanate (AITC); AFB_1_; Oriental mustard flour; yellow mustard flour; lactic acid bacteria

**Acknowledgments:** Ministry of Economy and Competitiveness (AGL2016-77610R) and the National Council for Scientific and Technological Development (CAPES/CNPq).

#### 6.4.4. Engineered Silver Nanoparticles, a Possible Tool in the Management of Aflatoxigenic and Ochratoxigenic Fungi and Aflatoxins and Ochratoxin A Production in Food

J.V. Gómez ^1^, A. Tarazona ^1^, E.M. Mateo ^2^, J. V. Gimeno-Adelantado ^3^, R. Mateo-Castro ^3^, M. Jiménez ^1^ and F. Mateo ^4,^*

^1^  Department of Microbiology and Ecology, University of Valencia, 46100 Burjassot, Valencia, Spain
^2^  Department of Microbiology, School of Medicine, University of Valencia, 46010 Valencia, Spain
^3^  Department of Analytical Chemistry, University of Valencia, 46100 Burjassot, Valencia, Spain
^4^  Department of Electronic Engineering, University of Valencia, 46100 Burjassot, Valencia, Spain

* Correspondence: Fernando.mateo@uv.es

**Abstract:** The most important and frequent mycotoxins in foods and feeds are aflatoxins (AFs) and ochratoxin A (OTA). These fungal secondary metabolites have strong detrimental impact on public health and economy. The main fungal species associated with AFs are *Aspergillus flavus* and *A. parasiticus*, and with OTA, *A. carbonarius*, *A. niger A. ochraceus, A. steynii*, *A. westerdijkiae* and *Penicillium verrucosum*. Nanotechnology is not new, in particular, nanoparticles have immense applications in agriculture, nutrition, medicine, health and other sciences. The potential use of nanotechnology in the control of toxigenic fungi and mycotoxin production has been little explored. In this report, engineered silver nanoparticles (AgNPs) were synthesized and characterized by transmission electron microscopy and single-particle inductively coupled plasma-mass spectrometry. Then, their effectiveness on control growth of all these fungal species and AF and OTA production by aflatoxigenic and ochratoxigenic species was studied. Spore suspensions supplemented with AgNPs (size 30 nm, range 14–100 nm) at doses 0–45 μg/mL were incubated for 2–30 h. At selected exposure times, the percentage of viable spores, effective doses (EDs) to inhibit the number of viable spores to 50%, 90% and 100% and fungal growth rates (GR) in maize-based medium (MBM) were estimated. AFs and OTA in cultures were determined by UPLC-ESI/MS/MS. Under the assayed conditions, EDs of AgNP, colony GR and AF and OTA levels in MBM cultures decreased when exposure time increased. EDs were higher for *A. flavus* and *A. parasiticus* than for ochratoxigenic species. The factors species, AgNP dose, exposure time and their interactions significantly affect fungal growth and AF and OTA accumulation in MBM. The results suggest that AgNPs alone or as active ingredient hosted in paints, films or other polymers could be a good strategy in the management of the main aflatoxigenic and ocratoxigenic species affecting food and AF and OTA contamination.

**Keywords:** silver nanoparticles; aflatoxins; ochratoxin A; effective doses; toxigenic fungi

**Acknowledgments:** The authors acknowledge financial support from the Ministry of Economy and Competitiveness (MINECO, Spanish Government) (Project AGL2014-53928-C2-1-R and Ph.D. contract BES-2015-071242).

**References**:

Gómez, J.V.; Tarazona, A.; Mateo, F.; Jiménez, M.; Mateo, E.M. Potential impact of engineered silver nanoparticles in the control of aflatoxins, ochratoxin A and the main aflatoxigenic and ochratoxigenic species affecting foods. *Food Control* **2019**, 101, 58–68.

## 7. Roundtable

*From feed to fork: safe food without mycotoxins*

*Chair: Ariane Vettorazzi. Universidad de Navarra*

Participants:

^1^  B. de Santis. Istituto Superiore di Sanità, Rome, Italy
^2^  F.J. Aldaz. Gobierno de Navarra, Navarra, Spain
^3^  M. Irurita. S.A.T. Lacturale, Navarra, Spain
^4^  J.M. Beunza. De Heus Nutrición Animal S.A.U, Navarra, Spain

## 8. Poster Communications

### 8.1. Does Pumpkin Extract Protect Mitochondria Against Mycotoxins? A Transcriptional Approach

M. Alonso-Garrido *, W. Zanned, G. Font and L. Manyes

Laboratory of Food Chemistry and Toxicology, Faculty of Pharmacy, University of Valencia, 46100 Burjassot, Valencia, Spain

* Correspondence: manuel.alonso-garrido@uv.es

**Abstract:** Pumpkin “Delica” *(Cucurbita maxima)* is well known for its high concentration on carotenoids, its dietary benefits and antioxidant activity. Mycotoxins are common toxins present in food and feed with an extended toxicity profile in humans and animals. Carotenoids and mycotoxins accumulate in a wide range of tissues and organs and both molecules possess the capability to penetrate the blood brain barrier. Since carotenoids protect against oxidation and mycotoxins have been reported to modify diverse cellular processes, human epithelial cells ECV 304 were selected as an in vitro model to analyze both cell viability (MTT) using nondifferentiated cells and gene expression (qPCR) using differentiated cells at day 9 and exposed for 2 h. Samples were: (a) non-treated (b) pumpkin extract (500 nM) treated cells, (c) treated cells only with mycotoxin mixtures (100 nM each mycotoxin) and (d) with pumpkin extract (500 nM) and mycotoxin mixtures (100 nM). Previous studies showed altered mitochondrial pathways due to mycotoxins, so key mitochondrial (MT-ND2, MT-ND3, MT-ND4, MT-ND4L, MT-ND5, MT-CO1, MT-ATP8) and nuclear (OSGIN1, RNR2) genes were chosen for analysis. The joint exposure to pumpkin extract and aflatoxins individually and combined did decreased cell viability at 24, 48 and 72h, while exposure to enniatins individually and combined did not show a tendency. Pumpkin extract treatment up-regulated all genes analyzed. In summary, conclusions are specific for each mycotoxin mixture and no pattern effect against the toxicity caused by these mycotoxins in ECV 304 could be found for pumpkin extract.

**Keywords:** qPCR; blood brain barrier; endothelial human cells ECV 304

**Acknowledgments:** This work was supported by the Spanish Ministry of Science, Innovation and Universities (AGL2016-77610-R and BES-2017-081328).

### 8.2. Silver Nanoparticles as Antifungal: Revision of Their Genotoxicity

A. Rodriguez-Garraus ^1,^*, A. Azqueta ^1,2^ and A. López de Cerain ^1,2^

^1^  Department of Pharmacology and Toxicology, School of Pharmacy and Nutrition, Universidad de Navarra, 31008 Pamplona, Spain
^2^  IdiSNA, Navarra Institute for Health Research, 31008 Pamplona, Spain

* Correspondence: arodriguez.53@alumni.unav.es

**Abstract:** Mycotoxins are secondary fungal metabolites that can be found as natural food contaminants in plants- and animal-derived products. The most important ones for human health are aflatoxins, produced by *Aspergillus* species, mainly *A. flavus* and *A. parasiticus*, ochratoxin, produced by *Penicillium* and *Aspergillus* species, and fumonisins, trichothecenes and zearalenones, synthesized by over 50 *Fusarium* species. Cereals, more specifically corn and wheat, are the main product affected by fungal contamination due to inadequate conditions pre-crop, during storage and post-harvest, limiting global crop production and crop quality. Antimicrobial agents used against mycotoxin producer fungi, can lead to resistant pathogens, and new fungicides are required in the field of agriculture to increase food availability, to reduce food waste and to increase food safety. In this regard, nanotechnology can provide new compounds, as silver nanoparticles, that have shown their antifungal activity and potency to disrupt mycotoxins production. Since the nineteenth century, silver-based compounds have been used in many antimicrobial applications, but there is a need to understand the risk that this material poses to human health. A bibliographic revision of 29 scientific articles has been made to batch and compare the silver nanoparticles genotoxicity results. From 49 comet, 15 FPG, 8 Endo-III and 1 OGG1 modified comet, 51 micronucleus and six mouse lymphoma in vitro assays, 40, 10, 6, 1, 31 and 6 showed significant genotoxic results, respectively. From 10 comet, 2 Endo-III and 2 OGG1 modified comet, 16 micronucleus, 2 Pig-a, 3 chromosome aberration, 2 DNA deletion and 4 HX2A determination in vivo assays, 4, 2, 2, 9, 0, 3, 2 and 3 reported positive genotoxic results, respectively. Smaller nanoparticles produce higher genotoxic effects. The results showed increased genotoxicity of nanoparticles coated with citrate compared to PVP. AgNPs accumulation in liver leads to long-term effects. Pig-a was shown to be inappropriate for the study of AgNPs genotoxicity.

**Keywords:** mycotoxins; silver nanoparticles; antifungal; genotoxicity

### 8.3. *Aspergillus welwitschiae* Isolated from Grapes and Raisins Produces Ochratoxin A

F.J. Cabañes *, M.L. Abarca and G. Castellá y M.R. Bragulat

Group de Micologia Veterinària, Departament de Sanitat i Anatomia Animals, Facultat de Veterinària, Universitat Autònoma de Barcelona, 08193 Bellaterra, Barcelona, Spain

* Correspondence: javier.cabanes@uab.cat

**Abstract:** Recently, the taxon *Aspergillus niger* sensu stricto has been split into *A. niger* and *A. welwitschiae* [1]. Both species cannot be distinguished by phenotypic or ecological data including extrolite profiles. So, this species has not yet been reported frequently because this old species name has been reintroduced not long ago [2]. In this study, two *A. niger* strains and five *A. welwitschiae* strains, isolated from wine grapes and raisins were studied. All the strains were confirmed for identity by sequencing of the calmodulin gene [3]. The aim of this study was to determine the effects of water activity (0.90; 0.95 and 0.98–0.99), culture media (Yeast Extract Sucrose Broth; Synthetic Grape Juice Medium; White grape juice) and temperature (15 °C, 25 °C and 35 °C) on the growth and OTA production of these strains. The assay was performed in microtiter plates, determining the absorbance at 530 nm and the concentration of OTA at 1, 2, 4 and 10 days. No significant differences were observed in absorbance and OTA production values between these two species. However, in this study, we have confirmed that *A. welwitschiae* strains from wine grapes and raisins are able to produce OTA.

**Keywords:***Aspergillus welwistchiae;* ecophysiology; grapes; ochratoxin A; raisins

**Acknowledgments:** This research was supported by the Ministerio de Economía y Competitividad of the Spanish Government (AGL2014-52516-R).

**References:**

1. Samson, R.A.; Visagie, C.M.; Houbraken, J., et al. Phylogeny, identification and nomenclature of the genus *Aspergillus*. *Stud. Mycol.* **2014**, *78*, 141–173.
2. Cabañes, F.J.; Bragulat, M.R. Black aspergilli and ochratoxin-producing species in foods. *Curr. Opin. Food Sci.* **2018**, *23*, 1–10.
3. Abarca, M.L.; Bragulat MRCabañes, F.J. Impact of some environmental factors on growth and ochratoxin A production by *Aspergillus niger* and *Aspergillus welwitschiae*. *Int. J. Food Microbiol.* **2019**, *291*, 10–16.

### 8.4. Effect of Allyl Isothiocyanate, Yellow Mustard Flour and Lactic Acid Bacteria Against *P. Verrucosum* Growth in Barley

K.C.P. Bocate ^1,^*, T.M. Nazareth ^2^, M.M. Navarro ^2^, M. Rivola ^3^, C. Luz ^2^, R. T. Caparrós ^2^, J. Quiles ^2^, J. Mañes ^2^ and G. Meca ^2^

^1^  Postgraduate Program in Animal Science, Pontifícia Universidade Católica do Paraná, 1155 Curitiba, Brasil
^2^  Laboratory of Food Chemistry and Toxicology, Faculty of Pharmacy, University of Valencia, 46100 Burjassot, Valencia, Spain
^3^  Department of Agriculture and Food Science, Alma Mater Studiorum, University of Bologna, 40127 Bologna, Italy

* Correspondence: karlabocate@gmail.com

**Abstract:** Allyl isothiocyanate (AITC) is one active ingredient of mustard essential oil and have shown antimicrobial action in previous studies. Yellow mustard is a Brassicaceae plant containing AITC, a volatile compound released as a result of mechanical damage to plant cells. It is studied for its antimicrobial, antifungal and mycotoxigenic capacity. Alternative measures are proposed to reduce the growth of mycotoxigenic fungi and the production of toxins, especially natural compounds, which receive better acceptance by consumers. As natural compounds, their use can attend the commercial demand for substitutes to chemicals traditionally used as preservatives in industry. This also applies to lactic acid bacteria (LAB) because some of their metabolites include preservative substances. Thus, the focus of this study was to evaluate the efficacy of AITC as a fumigant, yellow mustard flour (YMF) and lactic acid bacteria against the growth of *Penicillium verrucosum as* producer of ochratoxin A in barley. Different methods of treatment were applied by direct contact with the barley and by a modified atmosphere with different concentration of the compounds. The methods to analyse the efficacy of the compounds were minimum concentration inhibitory (MIC_50_), fungicide concentration (MFC), residual population, humidity and percentage of the germination. The results showed that in the concentration of 10 ppm of AITC, the fungal growth was reduced and the barley have capacity to germinate 85.8%. However, the treatments with LAB and the YMF did not show antifungal activity and reduced the capacity of the germination.

**Keywords:** natural compounds; antifungal; mustard flour; lactic acid bacteria

### 8.5. Effect of Essential Oils on the Production of Ochratoxin A by *Penicillium nordicum* in a Dry-Cured Fermented Sausages Model

E. Roncero *, M. Álvarez, M. A. Asensio, L. Sánchez-Montero, J. J. Rondán and M.J. Andrade

Food Hygiene and Safety, Meat and Meat Products Research Institute, Faculty of Veterinary Science, University of Extremadura, 10003 Cáceres, Spain

* Correspondence: eroncerob@unex.es

**Abstract:** One of the main hazards in dry-cured meat products is ochratoxin A (OTA), which is mostly produced by *Penicillium nordicum* and *Penicillium verrucosum.* Although antifungal compounds are currently applied on the surface of dry-cured fermented sausages to avoid the growth of undesirable moulds, there is an increasing trend to replace these chemical preservatives with others of natural origin. Within the latter, there are essential oils from aromatic plants whose effect against ochratoxigenic moulds of concern in cured meat products has been scarcely studied. The objective of this work was to evaluate the antagonistic activity of the essential oils obtained from three spices (rosemary, thyme and oregano) commonly added to dry-cured fermented sausages against ochratoxigenic moulds. For this, *P. nordicum* FHSCC Pn15 was inoculated in a dry-cured fermented sausage-based medium with and without the addition of different concentrations of each essential oil. As positive control, a batch with two concentrations of a commercial preparation with potassium sorbate and natamycin was prepared. After incubating at 12 °C for 15 days the mould growth was visually evaluated and OTA was quantified by uHPLC-MS/MS QqQ. Although no differences in the mould growth were detected among the treatments, OTA levels showed a significant increase with the addition of the highest concentration of the antifungal preparation. On the contrary, a significant decrease of OTA levels below the detection limit was observed when the rosemary essential oil was added at the highest concentration. Consequently, the use of rosemary essential oil could be considered an alternative strategy to commercial antifungal compounds to control the hazard posed by OTA in dry-cured fermented sausages.

**Keywords:***Penicillium nordicum;* dry-cured fermented sausages; ochratoxin A; antagonistic activity; essential oils

**Acknowledgments:** This work has been supported by IB16045 and GR18056 projects (Junta de Extremadura-Consejería de Economía e Infraestructuras-, Fondo Europeo de Desarrollo Regional-“Una manera de hacer Europa”). M. Álvarez is recipient of a pre-doctoral fellowship (BES-2017-081340) from the Spanish Ministry of Economy, Industry and Competitiveness.

### 8.6. Combined Toxicity of Aflatoxins and Ochratoxin A: A Systematic Review

M. Alonso-Jáuregui ^1,^*, A. López de Cerain ^1,2^, E. González-Peñas ^3^ and A. Vettorazzi ^1,2^

^1^  Department of Pharmacology and Toxicology, School of Pharmacy and Nutrition, Universidad de Navarra, 31008 Pamplona, Spain
^2^  IdiSNA, Navarra Institute for Health Research, 31008 Pamplona, Spain
^3^  Department of Pharmaceutical Technology and Chemistry, School of Pharmacy and Nutrition, Universidad de Navarra, 31008 Pamplona, Spain

* Correspondence: malonso.17@alumni.unav.es

**Abstract:** Mycotoxins are naturally occurring contaminants of food and feed. This is a public health concern due to human and animal exposure. Among them, aflatoxins (AFs) are considered as genotoxic carcinogens and no levels of exposure are considered as safe. Ochratoxin A (OTA) is a potent nephrotoxin and was classified as a possible human carcinogen. The current approach of toxicity evaluation and policies undertake the assumption of single exposure. However, the study of the combined toxicity resembles the real situation more effectively. The aim of the present work was to carry out a systematic review of the combined toxicity of AFs and OTA by gathering in vitro and in vivo studies as well as review articles. For that purpose, the PubMed database was used with the following keywords: aflatoxin AND ochratoxin AND toxicity. Scarce data were obtained. Although 234 records were identified, after applying exclusion criteria, 22 articles were finally analysed. All the studies selected evaluated AFB1 and OTA, except one article that analysed AFM1+OTA combined toxicity. On the one hand, the in vitro analysis (8 articles) of the combination resulted in cytotoxic additive effects in the cell lines representing the target organ of each mycotoxin (liver and kidney). In intestinal Caco-2 cells, a dual interaction was observed depending on the concentration tested. The binary mixture had no greater effects than OTA individually in MA-10 Leydig cells. Regarding immunotoxic potential, an increased response was observed in the combination. No conclusive results regarding genotoxicity have been obtained. On the other hand, the interaction in vivo (9 articles) turned out to be antagonistic for general and developmental toxicity, as well for in genotoxicity. However, regarding the immune response, an additive interaction was observed. Finally, 5 review articles were retrieved. The items explored were: co-occurrence at different levels (worldwide, EU and national), combined toxicity and experimental and statistical aspects for interaction characterization.

**Keywords:** aflatoxin; ochratoxin A; combined toxicity; mixture

**Acknowledgments:** This work was supported by the Spanish *Ministerio de Economía, Industria y Competitividad, Agencia Estatal de Investigación* (MultiMYCOtox project AGL2017-85732-R, MINECO/AEI/FEDER, UE). M.A.J. was supported by a grant from the *Asociación de Amigos* of the Universidad de Navarra, Spain.

### 8.7. Determination of Mycotoxins and Polyphenolic Compounds in Hemp Inflorescences Using UHPLC-Q-ORBITRAP HRMS

A. Narváez ^1,^*, L. Izzo ^1^, A. Gaspari ^1^, G. Graziani ^1^, A. Ritieni ^1^ and Y. Rodríguez-Carrasco ^2^

^1^  Department of Pharmacy, Università di Napoli Federico II, 80138 Napoli, Italy
^2^  Department of Food Chemistry and Toxicology, University of Valencia, 46100 Burjassot, Valencia, Spain

* Correspondence: alfonsonsimon@gmail.com

**Abstract:***Cannabis sativa* is a plant which produces a vast number of pharmacologically relevant compounds. Because of its therapeutic potential, *C. sativa* has been totally or partially legalized in countries like USA, Canada and others inside the EU. Alongside legalization, regulation about content in contaminants and therapeutic compounds has been set in only a few. Mycotoxin are toxic secondary metabolites produced by several fungi and *Fusarium* species have been reported as the third fungus more common in *C. sativa*. The most restrictive legislation (SOR/2018-144, Canada) sets limits for aflatoxins B1, B2, G1 and G2 and ochratoxin A, but *Fusarium spp.* can also produce different mycotoxins with a wide range of toxicological effects. Referring to therapeutic compounds, analyses are based on cannabinoids and terpenes, but *C. sativa* produces also polyphenolic compounds with biological activity reported. The aim of this study was to detect and quantify simultaneously different mycotoxins (*n = 19*) and polyphenolic compounds (*n = 42*) in hemp inflorescences throughout ultra-high performance liquid chromatography (UHPLC) coupled to Q-Orbitrap. Regulated mycotoxins were not found in any sample but beauvericin (*Fusarium* toxin) was quantified in four out of eight samples, in a range from 72.6 to 86.2 ng/g. These values are highly above the ones reported by several studies in grains and grain-based foodstuffs, and there are no maximum limits set by any legislation. Referring to polyphenols, 30 different compounds were found. The most important ones were the flavonoids luteolin, rutin and myricitin, which occurred in a wide range of concentrations, from 1 to 20 mg/g, whereas other phenolic compounds were quantified at concentrations below 1 mg/g. Since consumers use to have health issues, exposure to both regulated and non-regulated mycotoxins should be taking into account. Therefore, this procedure stands for a useful tool to control mycotoxins and therapeutical compounds in hemp.

**Keywords:** beauvericin; *Canabis sativa; Fusarium;* polyphenols; orbitrap

### 8.8. Mycotoxin Contamination and Fungal Populations in Silages for Dairy Cows in Spain

M. Rodríguez-Blanco, S. Marín, V. Sanchis and A.J. Ramos *

Applied Mycology Unit, Food Technology Department, University of Lleida, UTPV-XaRTA, Agrotecnio, 25198 Lleida, Spain

* Correspondence: ajramos@tecal.udl.es

**Abstract:** Ensiling is a practice commonly employed worldwide to preserve different kinds of crops for long periods of storage with similar nutritional values to the fresh materials. Since silages are one of the major components of the ruminant diet, these materials represent a potential source of mycotoxins as a consequence of the growth of filamentous fungi. The aim of this study was to analyse the presence of aflatoxins and *Fusarium* mycotoxins in different types of silages (maize, grass, alfalfa, sugar beet pulp, immature corn and ryegrass) collected in dairy farms located in four Spanish regions. Fungal populations, lactic acid bacteria, pH and water activity of the samples were also evaluated. *Penicillium* (4–26%), *Geotrichum* (2–21%) and *Monascus* (0.34–3%) were the main fungal genera identified in the microbiological survey. As for mycotoxins analysis, aflatoxins were found in 10% of the samples, being detected in samples of maize, alfalfa and immature corn silage. *Fusarium* mycotoxins were found in 40% of the analysed samples, and fumonisins (FBs) were the most commonly detected. These toxins were found in samples of maize, grass, alfalfa, sugar beet pulp and immature corn silage. Among the different types of silages studied, maize silage samples were the most heavily contaminated. Out of 44 analysed samples, 30 were contaminated by at least one mycotoxin: 41% were positive for the presence of FBs, 14% for deoxynivalenol, 23% for 15-acetyldeoxynivalenol and 16% for zearalenone. The levels of mycotoxins detected in the samples did not exceed the guidance values recommended by the EU. The lack of relationship between *Fusarium* counts in the microbiological study and the mycotoxin analysis pointed that these mycotoxins were probably synthetized before or immediately after ensiling. Mould growth and mycotoxin contamination in silages and crops, which are subjected to ensiling, should be regularly monitored in order to minimize the chronic exposure of dairy cows to mycotoxins through the intake of contaminated feed.

**Keywords:** silage; fungi; multi-mycotoxin analysis; UHPLC-FLD; HPLC-MS/MS

**Acknowledgments:** this work was supported by the Spanish Ministry of Economy and Competitiveness (project AGL2014-55379-P). María Rodríguez-Blanco thanks the University of Lleida for a pre-doctoral grant.

### 8.9. Single and Combined Actions of Ochratoxin A and Beauvericin in HEPG2 Cells: Viability, Micronucleus Induction and Cell Cycle Disturbance

A. Juan-García *, C. Juan, J. Tolosa and M.J. Ruiz

Laboratory of Food Chemistry and Toxicology, Faculty of Pharmacy, University of Valencia, 46100 Burjassot, Valencia, Spain

* Correspondence: ana.juan@uv.es

**Abstract:** Mycotoxins are produced by a number of fungal genera spp as e.g., *Aspergillus, Penicillium, Alternaria, Fusarium* and *Claviceps*. Beauvericin (BEA) and ochratoxin A (OTA) are present in various cereal crops and processed grains. For the first time, this study aims to determine their combination effect in HepG2 cells. In this study, the type of interaction among BEA and OTA through isobologram method, cell cycle disturbance by flow cytometry and, micronucleus induction (genotoxicity) by following the TG 487 (OECD, 2016) were studied. All three assay were performed individually and combined in HepG2 cells. Cytotoxic concentration ranges studied by the MTT assay, over 24, 48 and 72 h were from 0 to 25 µM for BEA; from 0 to 100 µM for OTA while BEA + OTA combinations at 1:10 ratio from 3.4 to 27.5 µM. The toxicity observed for BEA was higher than for OTA at all times assayed; additive and synergistic effects were detected for their mixtures. Cell cycle arrest in G0/G1 phase was detected for OTA and BEA + OTA treatment in HepG2 cells. Genotoxicity by MN induction revealed significant effects for BEA, OTA and in combinations underlining the importance of studying real exposure scenario of chronical exposure to mycotoxins.

**Keywords:** combination; micronucleus; viability; HepG2 cells

### 8.10. SH-SY5Y Cells Exposed to Ochratoxin A and Beauvericin: Comparison of Cytotoxic Effects

A. Juan-García *, F. Agahi and C. Juan

Laboratory of Food Chemistry and Toxicology, Faculty of Pharmacy, University of Valencia, 46100 Burjassot, Valencia, Spain

* Correspondence: ana.juan@uv.es

**Abstract:** The undifferentiated neuroblastoma cell line SH-SY5Y is a subclone of the SK-N-SH cell line derived from a bone marrow biopsy. It shares few properties with mature neurons; therefore, it is frequently used as a model to simulate the neuronal function and their differentiation. Fungal growth in human foods and animal feeds can cause formation of mycotoxins. Mycotoxins are known as secondary metabolites and main fungi genera producers belong to *Fusarium*, *Aspergillus* and *Penicillium*. The aim of this work is in one side to collect the studies and effects of various mycotoxin on SH-SY5Y; on the other side, to study the cytotoxic effects of beauvericin (BEA), and ochratoxin A (OTA) in that cell line. All articles selected address on in vitro cellular based assays. Cell viability has been studied by: propidium iodide assay, MTT (3-4,5-dimethylthiazol-2-yl]-2,5- diphenyltetrazolium bromide) assay, neutral red assay, lactate dehydrogenase leakage assay or reduction of the total cellular protein concentration. Here, cytotoxicity was measured by MTT assay, over 24, 48 and 72 h with a single treatment at the concentration range of 0.01 to 2.5 µM for bea and, from 0.2 to 50 µM for OTA at 1:2 dilutions. Based on the conversion of MTT into formazan crystal by cells that are alive, this assay determines mitochondrial activity and concentration that reach 50% inhibition of cellular proliferation (IC50). Individual IC50 values detected was diverse and BEA resulted to present higher toxic potential than OTA on SH-SY5Y cells.

**Keywords:** neuronal cells; Ochratoxin A (OTA); Beauvericin (BEA); MTT (3-4,5-dimethylthiazol-2-yl]-2,5-diphenyltetrazolium bromide); review

### 8.11. Control of Toxigenic Fungi Using Antigungal Proteins

J. Iribarren ^1^, J. Gil-Serna ^1,^*, A. Martínez del Pozo ^2^ and B. Patiño ^1^

^1^  Genetics, Physiology and Microbiology Department, University Complutense of Madrid, 28040 Madrid, Spain
^2^  Biochemistry and Molecular Biology Department, University Complutense of Madrid, 28040 Madrid, Spain

* Correspondence: jgilsern@ucm.es

**Abstract:** Crop contamination with toxigenic fungi, such as *Fusarium* spp. or *Aspergillus* spp. causes important economic and food nutritional value losses. Even considering this problem, it is not easy to find efficient methods to prevent mycotoxin production by these fungi. In this work, we tested crop-associated fungi against possible growth-inhibiting proteins with different origins: *Fusarium graminearum* Antifungal Protein (Fg-AFP) and Latrodectin-I (Ltd-I). The first one is produced by *Fusarium graminearum* to compete with other fungi, and the latter is found in the *Latrodectus hesperus* spider venom. A total of 10 strains of different *Fusarium* and *Aspergillus* species were tested against different concentrations of the purified proteins (7 µg/µL, 3.5 µg/µL, 1.4 µg/µL and 0.7 µg/µL) supplemented in Czapek agar plates incubated at 25 °C. Growth inhibition was observed in different concentrations on seven of the strains tested against Fg-AFP, finding a more significant inhibition with higher protein concentration. Ltd-I showed no effect in fungal growth, but it increased sporulation in eight strains, possibly indicating stress induction. Four isolates were used for the subsequent experiments included in *Aspergillus niger, A. flavus, Fusarium proliferatum* and *F. verticillioides*. They were also cultured in Czapek agar, including on-surface 100 µL of 1.5 µg/µL protein suspensions, confirming a growth reduction of 46.90%, 26.17%, 71.08% and 78.14%, respectively, with Fg-AFP, and no inhibition with Ltd-I. Production of aflatoxin B1 by *A. flavus* and ochratoxin A by *A. niger*, evaluated by Thin Layer Chromatography (TLC), was not affected by the proteins, and neither was the expression of *fum1* in *F. proliferatum* and *F. verticillioides*, evaluated by real-time reverse transcription polymerase chain reaction (RT-PCR).

**Keywords:** mycotoxins; antifungal protein; latrodectin-I; biological control; agrofood

**Acknowledgments:** This work was financed by the MINECO (AGL2014-53928-C2-2-R).

### 8.12. Mycotoxin Occurrence and Risk Assessment in Cereals from Algeria

C.K. Mahdjoubi ^1,2^, N. Arroyo-Manzanares ^3^, N. Hamini ^2^, A.M. García-Campaña ^1^ and L. Gámiz-Gracia ^1,^*

^1^  Department of Analytical Chemistry, Faculty of Sciences, University of Granada, 18071 Granada, Spain
^2^  Department of Biology, Faculty of Natural and Life Science, University of Oran 1, 31000 Oran, Algeria
^3^  Department of Analytical Chemistry, Faculty of Chemistry, University of Murcia, 30003 Murcia, Spain

* Correspondence: lgamiz@ugr.es

**Abstract:** The aim of this study was to evaluate the mycotoxin contamination of 120 samples of cereals (maize, rice, wheat and barley) from Algerian markets, considering that in this country there is no legislation about maximum allowed contents. With this purpose, a method using a QuEChERS-based extraction and ultra-high performance liquid chromatography coupled to tandem mass spectrometry (UHPLC–MS/MS) was developed. The target analytes included the mycotoxins regulated by the EU in cereals (aflatoxin B1, B2, G1 and G2, ochratoxin A, deoxynivalenol, zearalenone, fumonisin B1 and B2, T-2 and HT-2 toxin), other commonly studied mycotoxins (fusarenon-X, citrinin, sterigmatocystin) and emerging mycotoxins (beauvericin and enniatin A, A1, B and B1). Analytical results showed that 116 out of 120 total cereal samples (96%) were contaminated with at least one toxin. Aflatoxin G1 was the most frequently detected (69 samples, 57%) at concentrations up to 64 µg/kg. Moreover, fumonisins, zearalenone and deoxynivalenol registered very high concentrations, up to 49 mg/kg (fumonisin B1+B2), 0.58 mg/kg and 2.1 mg/kg, respectively, and in some maize samples the concentration of fumonisins exceeded in more than 10 times the maximum concentration allowed by EU. Remarkably, 57% of the samples were contaminated with emerging mycotoxins, and in 10 wheat samples the total concentration of emerging mycotoxins were between 2–10 mg/kg. Risk assessment was evaluated for all the mycotoxins with total dietary intake (TDI) available. The results showed that Algerian consumers are at a high risk of exposure to fumonisins (FB1+FB2) through maize (%TDI of 635.2) and to deoxynivalenol, zearalenone and the sum of HT-2 and T-2 toxins through wheat consumption (%TDI of 442, 234.6 and 231.5, respectively). These results advise Algerian consumers about the potential risk for mycotoxins.

**Keywords:** cereals; Algeria; QuEChERS (Quick, Easy, Cheap, Effective, Rugged and Safe); UHPLC-MS/MS; multimycotoxin

**Acknowledgments:** The authors gratefully acknowledge the financial support of the Spanish Ministry of Economy and Competitiveness (Project ref: AGL2015-70708-R, MINECO/FEDER, UE).

### 8.13. Evaluation of Aflatoxin Contamination in Cocoa Powder Samples

M. Herrera *, P. Alfonso, M. Biota, E. García-Rodríguez, S. Lorán, J.J. Carramiñana, T. Juan, A. Herrera and A. Ariño

Instituto Agroalimentario de Aragón-IA2, Veterinary Faculty, University of Zaragoza-CITA, 50013 Zaragoza, Spain

* Correspondence: herremar@unizar.es

**Abstract:** Aflatoxins pose a serious risk to food safety and cause high economic losses, contaminating crops in the field and during storage and affecting a wide variety of raw materials and processed foods. In addition, the incidence of these mycotoxins in the EU food supply chain has increased in recent years by factors related to the climate change. Foodstuffs contaminated with aflatoxins are a major threat to food safety, potentially affecting the most vulnerable population groups, such as children. Therefore, it is relevant to investigate the presence of aflatoxins in cocoa, which is frequently consumed by this high-risk population group. In addition, there is currently a certain legal gap in EU legislation since the content of aflatoxins B1, B2, G1 and G2, which are carcinogenic to humans (Group 1 of IARC), is not regulated for cocoa and derived products. The objective of this study was to evaluate the contamination by aflatoxins B1, B2, G1 and G2 in 50 commercial samples of branded cocoa powder (11 organic and 39 conventional). The mycotoxins were extracted with a methanol/water mixture (80:20) followed by cleanup using immunoaffinity columns. Finally, the determination was made by high-performance liquid chromatography coupled to photochemical (PHRED) and fluorescence (FLD) detectors, with a limit of detection of 0.02 μg/kg for each of the aflatoxins. The percentage of positivity to total aflatoxins was 44% (22 out of 50), with levels ranging from 0.02 μg/kg to 3.33 μg/kg. The incidence of the different aflatoxins was B1 (13 samples), G1 (11 samples) and B2 (3 samples); no aflatoxin G2 was detected. Aflatoxins B1, B2 and G1 were detected simultaneously in one sample, B1 and G1 in three samples, while B1 and B2 coexisted in two samples. The incidence of total aflatoxins was very similar in organic (45.5%) and conventional (43.6%) cocoa samples.

**Keywords:** mycotoxins; aflatoxins; cocoa; HPLC; infants and young children

**Acknowledgments:** Government of Aragón and FEDER 2014-2020 (Group A06_17R).

### 8.14. Antifungal Capacity of PTSO Against Micotoxigenic Strains of *Aspergillus Parasiticus*, *Aspergillus flavus*, *Penicillium Verrucosum* and *Fusarium Graminearum*

P. Abad ^1^, N. Arroyo-Manzanares ^2^, J.J. Ariza^1^, A. Baños ^1^, L. Gámiz-Gracia ^3^ and A.M. García-Campaña ^3,^*

^1^  DMC Research Center S.L.U., 18620 Alhendín, Granada, Spain
^2^  Department of Analytical Chemistry, Faculty of Sciences, University of Murcia, 30003 Murcia, Spain
^3^  Department of Analytical Chemistry, Faculty of Sciences, University of Granada, 18071 Granada, Spain

* Correspondence: amgarcia@ugr.es

**Abstract:** The organosulfur compounds characteristic of the allium genus, including propyl propane thiosulfonate (PTSO), are potentially useful antimicrobial compounds for food applications, including their application as feed additives. In this study, the antifungal activity of PTSO has been evaluated against different strains of aflatoxigenic fungi (*Aspergillus parasiticus* and *Aspergillus flavus*), a strain producer of ochratoxin A (OTA) (*Penicillium verrucosum*) and another zearalenone (ZEA)-producing strain (*Fusarium graminearum*) using an assay in culture medium. This study allowed to establish the lethal dose of PTSO against each of the studied strains, obtaining values of 10.0, 4.0, 7.5 and 17.5 mg/kg for *Penicillium verrucosum*, *Fusarium graminearum*, *Aspergillus parasiticus* and *Aspergillus flavus*, respectively. Likewise, considering that the production of mycotoxins is associated with the growth of fungi, in this study the influence of PTSO on the mycotoxin production has been also evaluated, using UHPLC-MS/MS for the determination of the mycotoxins under study. The results showed that, in addition to its antifungal activity, the PTSO also induces a reduction in the content of the mycotoxins ZEA and aflatoxin B2 (AFB2) produced by the fungi *Fusarium graminearum* and *Aspergillus flavus*, respectively. The concentration of ZEA was reduced by half when PTSO doses of 1.88 and 3.75 mg/kg were applied, while the content of AFB2 was reduced tenfold by using PTSO doses of 7.5 and 15 mg/kg. However, no change was observed in the concentrations of the other mycotoxins studied (OTA in *Penicillium verrucosum* and aflatoxin B1 (AFB1), aflatoxin G1 (AFG1), aflatoxin G2 (AFG2) and AFB2 in *Aspergillus parasiticus*).

**Keywords:** propyl propane thiosulfonate (PTSO); micotoxigenic strains; culture media; UHPLC-MS/MS

### 8.15. Evaluation of Mycotoxins During the Dry Process of *Triticum Aestivum* Silage from Tunisia

A. Mannai ^1^, S. Oueslati ^2^, H. Ben Salem ^1^, H. Berrada ^3^, J. Mañes ^3^ and C. Juan ^3,^*

^1^  Laboratory of Animal and Forage Productions, National Institute of Agronomic Research of Tunisia, (INRAT), 2049 Ariana, Tunisia
^2^  Regional Field Crop Research Center of Beja (CRRGC), 9000 Beja, Tunisia
^3^  Laboratory of Food Chemistry and Toxicology, Faculty of Pharmacy, University of Valencia, 46100 Burjassot, Valencia, Spain

* Correspondence: cristina.juan@uv.es

**Abstract:** The incidence of *Fusarium* mycotoxins has proved interesting for assessing the quality of silage. The aim of this work was study the incidence of 23 mycotoxins in a set of 18 *Triticum aestivum* silage samples, obtained from two different geographical regions in the Northern Tunisia (Bizerte and Ariana) during four periods of the dried silage procedure. Mycotoxins were separated and purified from samples by a liquid–solid extraction procedure using C_2_H_3_N/H_2_O/CH_3_COOH (79:20:1, v/v/v) and determined with liquid chromatography coupled to a triple quadrupole mass spectrometry (LC-MS/MS). Presence of deoxynivalenol (DON), enniantin A1, B, B1 (ENA1, ENB, ENB1), HT-2 toxin, beauvericin (BEA), and zearalenone ZEA were detected in the analyzed samples at the following percentages: 29%, 29%, 12%, 12%, 6%, 6% and 6%. DON and ENB were the most detected mycotoxin in wheat silage samples with mean values of 40 ng/kg, and 1987 µg/kg, respectively. The most contaminated samples were detected during the first and fourth period of dried silage process in both regions. However, the Ariana cultivated silage was the most contaminated, in fact 58% of them presented mycotoxins and the majority contamination was associated to ENB (33%). These results suggested that DON and ENB are the most field mycotoxins found after the processes of fermentation in *Triticum aestivum* silage.

**Keywords:** deoxynivalenol; nivalenol; *Triticum aestivum* silage; LC-MS/MS

**Acknowledgments:** AGL2016-77610-R.

### 8.16. Emerging Mycotoxins in Barley from Tunisia: Results of Four Years Study (2015–2018)

S. Oueslati ^1,^*, H. Berrada ^2^, J. Mañes ^2^ and C. Juan ^2^

^1^  Regional Field Crop Research Center of Beja (CRRGC), 9000 Beja, Tunisia
^2^  Laboratory of Food Chemistry and Toxicology, Faculty of Pharmacy, University of Valencia, 46100 Burjassot, Valencia, Spain

* Correspondence: souheibo@yahoo.fr

**Abstract:** Tunisia, as well as other countries across the Mediterranean, is suffering from the climate changes and it is necessary to develop strategies to guarantee the availability and the quality of staple foods for its population, such as cereals. These changes are stimulating, among other biocaontaminants, toxigenic fungi, which are able to produce a group of emerging mycotoxins mainly enniatins (ENA, ENA1, ENB, and ENB1) as well as beauvericin (BEA). In this study, the natural occurrence of ENA, ENA1, ENB, ENB1 and BEA, in the main cultivated barley varieties intended for human consumption was assessed. The varieties samples termed Manel, Kounouz, Rihane, Lemsi were cultivated in the same area, and the same agronomic conditions were performed all along. Samples were collected in the Northern Tunisia (region of Béja) and the monitoring was realized along four successive years (2015–2018). The collected samples were extracted using a liquid–solid extraction using a mixture of acetonitrile:water (84:16, v/v) prior to a liquid chromatography-tandem mass spectrometry determination. Results showed the presence of both ENB and ENB1 in the Manel sample collected in 2017. Values reached 46.6 and 5.4 µg/Kg for ENB and ENB1, respectively. While all the other samples of each studied year were not contaminated by the analyzed mycotoxins. To the best of our knowledge, this is the first survey of Tunisian barley varieties contamination by the emerging mycotoxins for a period of consecutive four years. Even though meteorological conditions may be favorable for the growth of toxigenic fungi—mainly *Fusarium avenaceum* in our case—the varietal resistance itself and the agronomic pack performed within the field were able to insure a production with no toxicological issues. Such results may be used in national breeding programs in order to continue improving barley varieties to fight against climate changes.

**Keywords:** enniatin; beauvericin; LC-MS/MS; barley; Tunisia

**Acknowledgments:** AGL2016-77610-R.

### 8.17. Biocontrol Potential of Acid Lactic Bacteria Against Mycotoxigenic Fungi in Oranges

V. D’Opazo*, C. Luz, R. Torrijos, T. Nazareth, J. Quiles, J. Mañes and G. Meca

Laboratory of Food Chemistry and Toxicology, Faculty of Pharmacy, University of Valencia, 46100 Burjassot, Valencia, Spain

* Correspondence: vicdota@alumni.uv.es

**Abstract:** Due to the increasing trend of reducing the use of synthetic compounds in food, the application of safer and older preservation techniques is being recovered. Those old techniques are known to be safe because of their long history of use. Fermentation has been one of the most used preservation methods, and may prove to be a good alternative to the methods used nowadays. Following the tendency in this study, the antifungal activity from nine strains of acid lactic bacteria were tested against mycotoxygenic fungi of different genera, *Fusarium, Penicillium*, and *Aspergillus,* using agar diffusion, overlay and MIC/MFC (minimum inhibitory concentration, minimum fungicidal concentration) methods. Furthermore, a determination of the principal volatile organic compounds (VOC) from medium fermented for 72 h was performed in a CG-MS. Finally, the fermented mediums with the lower MIC/MFC were used to treat oranges inoculated with *Penicillium expansum* and *Penicillium digitatum* in order to test theirs biopreservation potential. This treatment was carried out by spraying the fermented medium over the fermented oranges, with a concentration of 25g/L. The results of the antifungal activity test shown MIC from 1.6–100 mg/mL and MFC from 3.1–100 mg/mL, showed that *Aspergillus* were the most resistant and *Fusarium* the most susceptible to fermented mediums. Pyrazines were main detected VOC in the fermented MRS-B, conforming to 39–75% of the total. The results of the food tests showed a reduction of the growth of *P. expansum* in treated oranges.

**Keywords:** biopreservation; toxigenic fungi; lactic acid bacteria

### 8.18. Control Growth of *Aspergillus Steynii* and Ochratoxin A Biosynthesis by Bioactive ethylene-vinyl alcohol copolymer EVOH Films Containing Essential Oils

A. Tarazona ^1^, J.V. Gómez ^1^, E.M. Mateo ^2^, R. Gavara ^3^, R. Mateo-Castro ^4^, José V. Gimeno-Adelantado ^4^, M. Jiménez ^1^ and F. Mateo ^5,^*

^1^  Department of Microbiology and Ecology, University of Valencia, 46100 Burjassot, Valencia, Spain
^2^  Department of Microbiology, School of Medicine, University of Valencia, 46010 Valencia, Spain
^3^  Packaging Lab, Institute of Agrochemistry and Food Technology, CSIC, 46980 Paterna, Valencia, Spain
^4^  Department of Analytical Chemistry, University of Valencia, 46100 Burjassot, Valencia, Spain
^5^  Deparment of Electronic Engineering, University of Valencia, 46100 Burjassot, Valencia, Spain

* Correspondence: Fernando.mateo@uv.es

**Abstract:***Aspergillus steynii* is possibly the main ochratoxin A (OTA) producing species in food and feed. OTA is a potent nephrotoxic, teratogenic, embryotoxic, genotoxic, neurotoxic, carcinogenic and immunosuppressive compound. Cereals are the main OTA source in the diet. The aim of this study was to develop effective bioactive films for controlling *A. steynii* growth and OTA production in maize grains and, by extension, in other possible food and feed. To this purpose i) bioactive ethylene-vinyl alcohol copolymer (EVOH) films incorporating cinnamaldehyde (CINHO), linalool (LIN), isoeugenol (IEG) or citral (CIT) (considered in the GRAS category) were prepared, ii) the ability of the designed active films to reduce/inhibit the growth of *A. steynii* in maize grains under different environmental conditions was determined, and iii) the effect of these active films to inhibit OTA accumulation in the seeds under the assayed conditions was tested. ANOVA showed that film class, a_w_, temperature and their interactions significantly affected growth rates and OTA production. The most effective films were those containing CINHO. ED_50_, ED_90_ and ED_100_ (Minimum lethal concentration, MLC) ranged 165–350, 297–601, 333–666 μg EVOH-CINHO/25 g maize grains, respectively, depending on environmental conditions. The least efficient were EVOH-LIN films, since the ED_50_, ED_90_ and ED_100_ were 2800–>3330, >3330 and >3330 μg EVOH-LIN/25 g maize grains, respectively. The effectiveness of bioactive films increased with increasing doses. Optimal fungal growth and OTA production happened at 32 °C. This species can be very competitive in warm climates and under storage conditions. The EVOH-CINHO films followed by EVOH-IEG and EVOH-CIT films, designed in this study can be potent antifungal agents against *A. steynii* and strong inhibitors of OTA biosynthesis in maize grains at very low doses. This is the first study on the impact that interacting environmental conditions and bioactive films have on the growth of *A. steynii* and OTA production.

**Keywords:***Aspergillus steynii;* bioactive films; essential oils; effective doses; ochratoxin A

**Acknowledgments:** The authors acknowledge financial support from the Ministry of Economy and Competitiveness (MINECO, Spanish Government) (Project AGL2014-53928-C2-1-R and Ph.D. contract BES-2015-071242)/.

**References**

Tarazona, A.; Gómez, J.V.; Gavara, R.; Mateo-Castro, R.; Gimeno-Adelantado, J.V.; Jiménez, M.; Mateo, E.M. Risk management of ochratoxigenic fungi and ochratoxin A in maize grains by bioactive EVOH films containing individual components of some essential oils. *Int. J. Food Microbiol.* **2018**, *269*, 107–119.

### 8.19. Prediction by Neural Networks of Ochratoxin A Biosynthesis by *Aspergillus Steynii* and *A. Carbonarius* in Selected Media Supplemented with Ecological and Non-Ecological Fungicides

E.M. Mateo ^1,^*, A. Tarazona ^2^, J.V. Gómez ^2^, M.A. García-Esparza ^3^, G.V. Gimeno-Adelantado ^4^ and F. Mateo ^5,^*

^1^  Department of Microbiology, School of Medicine, University of Valencia, 46010, Valencia, Spain
^2^  Department of Microbiology and Ecology, University of Valencia, 46100 Burjassot, Valencia, Spain
^3^  Departmente of Pharmacy, School of Health Sciences, University Cardenal Herrera CEU, 46115 Alfara del Patriarca, Valencia, Spain
^4^  Department of Analytical Chemistry, University of Valencia, 46100 Burjassot, Valencia, Spain
^5^  Department of Electronic Engineering, University of Valencia, 46100 Burjassot, Valencia, Spain

* Correspondence: eva.mateo@uv.es

**Abstract:** Toxigenic fungi and mycotoxins cause devastating effects on agricultural crops, economy, food security and human and animal health. Ochratoxin A (OTA) is a potent nephrotoxin also known to be teratogenic, immunosuppresive and carcinogenic. In Spain, *Aspergillus steynii* and *A. carbonarius* are the main ochratoxigenic species in cereals and grapes, respectively. Copper oxychloride and sulfur are non-systemic fungicides, widely used against many fungal diseases, especially in ecological agriculture. Mancozeb is a broad range contact non-selective fungicide but it is not authorized in organic farming. The aims of this study were: (i) to assay the effect of these fungicides on OTA biosynthesis by *A. steynii* and *A. carbonarius* isolates from Spanish oat grains and grapes, respectively, under different temperatures and (ii) to study the possibility of using artificial neural networks (NNs) encompassing both multilayer perceptrons (MLP–NN) and radial-basis function networks (RBFN) to predict OTA accumulation over time in cultures. Oat-based agar medium and grape-based agar medium were used for *A. steynii* and *A. carbonarius cultures*, respectively. Media were supplemented with mancozeb (1–30 mg/L), copper oxychloride (5–500 mg/L) and sulfur (10–8000 mg/L). Incubation temperatures were 15 and 25 °C. OTA determination was performed by UPLC–ESI/MS/MS. MLP–NN with 1/2 hidden layers (*HL*) of *N* nodes were tested to predict as accurately as possible OTA levels in cultures. Temperature, fungicide-dose and time (inputs) significantly influenced OTA concentration (output) in cultures. Sub-inhibitory doses of these antifungal agents can enhance OTA production. Mancozeb proved more effective than ecological fungicides to prevent OTA production at similar doses. Good MLP–NN topologies for both fungi were 3:26:1 or 3:24:1 (1 *HL*) and 3:14:12:1 or 3:16:10:1 (2 *HL*) without validation. With validation, MLPs with the same topologies showed slightly higher MSEtest, but the overtraining drawbacks can be avoided. RBFN with 60–65 nodes proved also useful.

**Keywords:***Aspergillus steynii; A. carbonarius*; neural networks; ochratoxin A; antifungal agents

**Acknowledgments:** The authors acknowledge financial support from the Ministry of Economy and Competitiveness (MINECO, Spanish Government) (Project AGL2014-53928-C2-1-R and Ph.D. contract BES-2015-071242).

### 8.20. Predictive Models for Deoxynivalenol Biosynthesis by *Fusarium Culmorum* in Maize Grains Under Different Environmental Conditions

F. Mateo ^1,^*, A. Tarazona ^2^, J.V. Gómez ^2^, G.V. Gimeno-Adelantado ^3^, D. Romera ^2^ and E.M. Mateo ^4^

^1^  Department of Electronic Engineering, University of Valencia, 46100 Burjassot, Valencia, Spain
^2^  Department of Microbiology and Ecology, University of Valencia, 46100 Burjassot, Valencia, Spain
^3^  Department of Analytical Chemistry, University of Valencia, 46100 Burjassot, Valencia, Spain
^4^  Department of Microbiology, School of Medicine, University of Valencia, 46010 Burjassot, Valencia, Spain

* Correspondence: fernando.mateo@uv.es

**Abstract:** Deoxynivalenol (DON) is produced mainly by *Fusarium culmorum* and *F. graminearum* in cereal crops worldwide. Pathogen surveys indicate that *F. culmorum* is the primary etiological agent of *Fusarium* crown rot in Mediterranean countries. DON affects emetic response, growth, immune function and reproduction. Accurate forecasting of DON accumulation in cereals is of great interest although many factors affect its biosynthesis. The possibility of using artificial neural networks (NNs) encompassing both multilayer perceptron (MLP-NN) and radial-basis function networks (RBFN) to predict DON accumulation over time in maize grain cultures of *F. culmorum* has been tested. The input variables were temperature, a_w_, time and fungal contamination level. The output was DON concentration. For MLP-NN development, a data subset was used for training/building models. Another subset was used (or omitted) for hold-out validation and another one, not used for training, served to test the NN. No validation subset is needed for RBFN. The criterion for model optimization was minimizing the mean square error (MSE) for test. Models were developed using the NN toolbox of MATLAB. DON levels depended on all the input variables. NNs can predict the behavior of the strain regarding DON accumulation when the input variables are shown to the networks. Prediction accuracy depended on NN type. For MLPs, the training algorithm and the fact of using/omitting validation conditioned the number of hidden nodes (*N*) for the optimum architecture. A MLP-NN with one hidden layer without validation gave the minimum MSE_test_ among the MLPs. The optimum architecture implied *N* = 8 hidden nodes. When validation was applied, the best MLP had two hidden layers with *N*_1_ = *N*_2_ = 12. A RBFN with *N* = 80 nodes produced the lowest MSE_test_ and proved the best network with a R_2_ = 0.978 for predicted vs. observed DON level plot. Thus, accurate predictability of DON level in vitro can be attained using NNs.

**Keywords:** predictive models; artificial neural networks; *F. culmorum*; deoxynivalenol (DON); maize; climatic change

**Acknowledgments:** The authors acknowledge financial support from the Ministry of Economy and Competitiveness (MINECO, Spanish Government) (Project AGL2014-53928-C2-1-R and Ph.D. contract BES-2015-071242).

### 8.21. Comparison of the Toxic Effects of Sterigmatocystin in the HEPG2 and SH-SY5Y Cell Lines

V. Zingales *, M. Fernández-Franzón and M.J. Ruiz

Laboratorio de Toxicología, Facultat de Farmàcia, University of Valencia, 46100 Burjassot, Valencia, Spain

* Correspondence: vezin@uv.es

**Abstract:** Sterigmatocystin (STE) is a mycotoxin produced mainly by fungi belonging to the genus *Aspergillus*. Taking into account the hepatocarcinogenic properties of STE and the limited number of studies on its toxicity on the nervous system, the aim of the present study was to evaluate the toxic effects produced by STE in human hepatocarcinoma (HepG2) and in human neuroblastoma (SH-SY5Y) cells. To carry out this objective, the effects of STE on cell viability, levels of reactive oxygen species (ROS) and alteration of mitochondrial membrane potential (MMP) were investigated. The concentration used to evaluate the effects on cell viability ranged from 0.78 to 50 μM. STE decreased cell proliferation depending on the concentration and time of exposure in both cell lines. However, IC_50_ values were not obtained in the HepG2 cells at any of the exposure times tested (24, 48 and 72h). About the SH-SY5Y cells, only IC_50_ values were obtained at the highest exposure times, 48h (2.12 ± 2.04) and 72h (0.52 ± 0.08). To determine the mechanism of toxicity of STE, both cell lines were exposed for 24 h at 0.78; 1.56 and 3.12μM of STE. The results showed that STE did not produce ROS in any of the cell lines used. However, a significant alteration of the MMP was observed at the highest concentration tested in the HepG2 cells. In conclusion, the results show that STE is more cytotoxic for neuronal cells than hepatic cells. The toxic effect does not seem to be related to the early production of ROS in both cell lines, while it could be related to the alteration of MMP in HepG2 cells. However, other mechanisms of action should be investigated

**Keywords:** sterigmatocystin; cytotoxicity; oxidative stress; mitochondrial membrane potential; SH-SY5Y; HepG2 cells

**Acknowledgments:** Ministry of Economy and Competitiveness (AGL 2016-77610-R) and the pre-doctoral research training program “Programa Santiago Grisolia (GRISOLIAP/2018/092) CPI-18-117”.

### 8.22. Application of Natamycin in Products Affected by Toxigenic Fungi

R. Torrijos ^1,^*, J. Pérez ^1^, T.M. Nazareth ^1,2^, J.M. Quiles ^1^, C. Luz ^1^, J. Mañes ^1^ and G. Meca ^1^

^1^  Departamento de Medicina Preventiva, Facultad de Farmacia, University of Valencia, 46100 Burjassot, Valencia, Spain
^2^  School of Life Science, Pontifícia Universidade Católica do Paraná, 1155 Curitiba, Brasil

* Correspondence: Raquel.Torrijos@uv.es

**Abstract:** Natamycin is a macrolide polyene produced by the fermentation of the *Streptomyces natalensis* bacteria. The objective of the study was the evaluation of natamycin as an antifungal agent against multiple strains belonging to the *Aspergillus*, *Penicillium* and *Fusarium* genera, establishing the minimum inhibitory concentration (MIC) and the minimum fungicidal concentration (MFC). Three strategies were studied for products commonly affected by toxigenic fungi. The first strategy evaluated the application of natamycin by spraying at 0.25, 0.5 and 1 mg/dm2 on the surface of mozzarella cheese slices contaminated with *P. commune* CECT 20767. The following strategy studied the incorporation of natamycin at 0.25, 0.5 and 1 mg/dm2 in hydroxyethylcellulose biofilms (HEC) 2% (w/v) and glycerol (0.5% w/v) in the same product. The last strategy evaluated the application of natamycin by spraying contaminated corn with *F. graminearum* ITEM 126 at doses of 25, 50 and 100 μg/g, determining the content of mycotoxins at 20 and 40 days of incubation by LC-ESI/MS Q-TOF. Natamycin was effective against all strains tested. The strategies evaluated for the reduction of the fungal population of *P. commune* in mozzarella cheese were effective, increasing the shelf life of the product. Three mycotoxins (Fumonisin B1, Neosolaniol and Fusarenon X) and two metabolites (Deoxynivalenol-3-glucoside, T2-triol) were detected in corn at 40 days of incubation. The dose of 100 μg/g of natamycin reduced the presence of these mycotoxins between 31.7 and 77.1%, while for the rest of mycotoxins a complete inhibition was achieved. Natamycin has shown to be a substance of interest in the application of products commonly affected by toxigenic fungi. This substance could be an alternative to the use of synthetic chemical additives.

**Keywords:** antifungal activity; biofilm; cheese; maize; natamycin

**Acknowledgments:** The research was supported by the Spanish Ministry of Economy and Competitiveness (AGL2016-77610R), the project Prometeo (2018/216) and by the pre-PhD program of the Spanish Ministry of Science, Innovation and Universities (FPU17/06104).

### 8.23. Use of Essential Oils as A Green Potential Alternative Against Mycotoxigenic Fungi

G. Pronesti ^1^, K.C.P. Bocate ^2^, C. Luz ^3^, G. Meca ^3^ and Y. Rodríguez-Carrasco ^3,^*

^1^  Faculty of Pharmacy, University of Camerino, 62032 Camerino, Italy
^2^  Postgraduate Program in Animal Science, Pontifícia Universidade Católica do Paraná, 1155 Curitiba, Brasil
^3^  Laboratory of Food Chemistry and Toxicology, Faculty of Pharmacy, University of Valencia, 46100 Burjassot, Valencia, Spain

* Correspondence: yelko.Rodriguez@uv.es

**Abstract:** Essential oils (EOs) are natural compounds biosynthesized in different plant organs and obtained by hydrodistillation. EOs are employed in food industries for their antifungal and antimycotoxigenic properties against pathogenic fungi which can colonize foodstuffs. Thus, the aim of this work is to evaluate the inhibition activity of several EOs from different plant families against six different mycotoxigenic fungi belonging to *Aspergillus*, *Fusarium* and *Penicillium* genera. minimal inhibitory concentration (MIC) and minimal fungicidal concentration (MFC) were investigated. EOs from *Apiaceae*, *Lamiaceae* and *Asteraceae* families showed the lower values ranging from 75 ppm and 1200 ppm for the MIC50 and from 300 ppm and 2500 ppm for the MFC. Carlina radix (*Asteraceae*) and Thymus capitatus (*Lamiaceae*) showed the lowest values MIC50 (75 ppm) and MFC (600 ppm) against *P. verrucosum*. These results suggest that many EOs are able not only to inhibit, but also to get rid the most significant mycotoxigenic fungi which are able to release important compounds from a toxicological point of view. These preliminary in vitro data promote future studies for considering EOs as a potential food preservatives. However, changes in their organoleptic properties due to the usage of these volatile compounds must be studied further.

**Keywords:** essential oils; antifungal; antimycotoxigenic; minimal inhibitory concentration (MIC50); minimal fungicidal concentration (MFC)

### 8.24. Purification and Identification of Peptides Obtained from the Fish Industry Byproduct Fermentation

C. Luz *, A. Tornos, A. Princep, F. Barba, J. Mañes and G. Meca

Laboratorio de Química de los Alimentos y Toxicología, Facultat de Farmàcia, University of Valencia, 46100 Burjassot, Valencia, Spain

* Correspondence: Carlos.Luz@uv.es

**Abstract:** The fish industry generates a large amount of processing byproducts, representing between 30 and 70% of the weight of the raw material. In the case of sea bass, the main waste consists of head, viscera, spines and skin. The industry’s interest in seeking new use potential for these byproducts and revaluing them is increasing. The objective of the present study was to isolate lactic acid bacteria from sea bass to be used in the development of fermentative processes and to obtain bioactive antimicrobial compounds that increase the value of these byproducts. Then, bacteria from the stomach, intestine and colon of sea bass were isolated by culture in de man, rogosa and sharpe (MRS) agar at 37 °C in anaerobiosis. With the isolated bacteria, two types of broths were fermented; waste broth, made with fish byproducts and meat broth, made with fish fillets. After inoculation of the bacteria and 72 h of incubation at 37 °C, the fermented broth obtained was lyophilized to concentrate the sample. Qualitative and quantitative studies of antifungal activity were performed against a selection of toxigenic fungi, as well as antioxidant activity by the 2,2’-azino-bis(3-ethylbenzothiazoline-6-sulphonic acid (ABTS) method. Next, we carried out a study of the protein profile of the broths using sodium dodecyl sulfate–polyacrylamide gel electrophoresis (SDS PAGE). The broths fermented by the most proteolytic bacteria were selected to perform a purification and identification by gel filtration chromatography and analysis by Liquid Chromatography-Electrospray-Mass Spectrometry-Time of Flight (LC-ESI-MS-TOF) of the peptides generated from the fermentation. The results showed antifungal activity against all the studied genera and greater antioxidant activity than the non-fermented control broth. Analysis by MS-TOF and data processing using the Spectrum Mill software identified a series of peptides derived from the hydrolysis of sea bass proteins.

**Keywords:** antifungal activity; revaluation of byproducts; toxigenic fungi; fish; peptide sequencing

**Acknowledgments:** This research study was supported by the Ministry of Economy and Competitiveness (AGL2016-77610R), by Generalitat Valenciana Prometeo/2018/126 and by the Universitat de València with PhD program of “Atracció de Talent (4690/4690)”.

## Figures and Tables

**Table 1:** Research groups in the MICOFOOD network.

Name	University	Contact	E-mail	Web Site
Agrifood Fungi and Yeast	Complutense of Madrid	C. Vázquez	covi@bio.ucm.es	https://www.ucm.es/hongos-y-levaduras/
Applied Mycology	Lleida	V. Sanchís	vsanchis@tecal.udl.cat	http://www.tecal.udl.cat/export/sites/Tecal/en/recerca/Grups/Informacion-grupo-Micologia-Aplicada-english.pdf
Food Chemistry and Toxicology (COAL)	Valencia	J. Mañes	Jordi.Manes@uv.es	https://www.uv.es/uvweb/departamento-medicina-preventiva-salut-publica-ciencies-alimentacio-toxicologia-medicina-legal/es/investigacion/grupos-investigacion/contaminantes-alimentos/presentacion-1285859599236.html
Food Hygiene and Safety, Meat and Meat Products Research Institute	Extremadura	M. Rodríguez	marrodri@unex.es	http://higiene.unex.es
FQM-302-Quality in Food, Environmental and Clinical Analytical Chemistry	Granada	A. M. García-Campaña	amgarcia@ugr.es	https://www.ugr.es/~fqm302/
Laboratory of Hygiene, Inspection and Control of Foods	Santiago de Compostela	A. Cepeda	alberto.cepeda@usc.es	http://imaisd.usc.es/grupoficha.asp?idpersoatipogrupo=75517&i=es&s=-126-191-196-235
Mycology and Mycotoxins	Valencia	M. Jiménez	Misericordia.jimenez@uv.es	http://www.uv.es/biologia – http://www.uv.es/quimica
Mycotoxin Research Group of the University of Zaragoza (MYCOZAR)	Zaragoza	A. Ariño	aarino@unizar.es	http://micofood.es/mycozar-grupo-de-investigacion-en-micotoxinas-de-la-universidad-de-zaragoza/
Postharvest Fungal Pathogens and Food Spoilage Fungi Lab	IATA (CSIC)*	L. González-Candelas	lgonzalez@iata.csic.es	http://w1.iata.csic.es/penipato
Research Group on Mycotoxins (MITOX)	Navarra	A. López de Cerain E. González-Peñas	acerain@unav.es mgpenas@unav.es	https://www.unav.edu/en/web/research-on-mycotoxins
Veterinary Mycology Group	Autònoma de Barcelona	F. J. Cabañes	Javier.Cabanes@uab.cat	http://sct.uab.cat/svbm/content/grup-de-micologia-veterin%C3%A0ria

